# Nutritional Gaps and Supplementation in the First 1000 Days

**DOI:** 10.3390/nu11122891

**Published:** 2019-11-27

**Authors:** Katrina Beluska-Turkan, Renee Korczak, Beth Hartell, Kristin Moskal, Johanna Maukonen, Diane E. Alexander, Norman Salem, Laura Harkness, Wafaa Ayad, Jacalyn Szaro, Kelly Zhang, Nalin Siriwardhana

**Affiliations:** 1 Church & Dwight, Co., Inc., Product Development Nutritional Sciences, Princeton, NJ 08540, USA; Katrina.Beluska-Turkan@churchdwight.com (K.B.-T.); Kristin.Moskal@churchdwight.com (K.M.); Laura.Harkness@churchdwight.com (L.H.); wafaa.ayad@churchdwight.com (W.A.); szaro0607@gmail.com (J.S.); kelly.zhang@churchdwight.com (K.Z.); 2 Premier Nutrition, LLC, Bernardsville, NJ 07924, USA; renee@premierdietitian.com; 3 PearTree Nutrition, LLC, Seattle, WA 98115, USA; beth.hartell@peartreenutrition.com; 4 Dupont Nutrition & Biosciences, FIN-02460 Kantvik, Finland; pia-johanna.maukonen@dupont.com; 5 Kemin Industries, Inc., Des Moines, IA 50317, USA; dee0408@gmail.com; 6 DSM Nutritional Products, Columbia, MD 21045, USA; Norman.Salem@dsm.com

**Keywords:** first 1000 days, nutrition, deficiency, supplementation, life cycle, pregnancy, early childhood

## Abstract

Optimized nutrition during the first 1000 days (from conception through the 2nd birthday) is critical for healthy development and a healthy life for the newborn. Pregnancy and the postpartum period are accompanied by physiological changes, increased energy needs, and changing requirements in the nutrients critical for optimal growth and development. Infants and toddlers also experience physiological changes and have specific nutritional needs. Food and nutrition experts can provide women of childbearing age with adequate dietary advice to optimize nutrition, as well as guidance on selecting appropriate dietary supplements. Considering the approaching 2020–2025 Dietary Guidelines for Americans (DGA) will be making specific recommendations for children, it is important to provide accurate scientific information to support health influencers in the field of nutrition. The purpose of this review is to summarize the nutrition and supplementation literature for the first 1000 days; to highlight nutritional and knowledge gaps; and to educate nutrition influencers to provide thoughtful guidance to mothers and families. Optimal nutrition during pregnancy through early childhood is critical for supporting a healthy life. Nutrition influencers, such as dietitians, obstetricians/gynecologists, and other relevant health professionals, should continue guiding supplement and food intake and work closely with expectant families and nutrition gatekeepers.

## 1. Introduction

The first 1000 days of early life refers to the period from conception through the child’s second birthday [1]. Optimal nutrition during this time is essential for supporting critical periods of fetal growth and development, maternal health (including the postpartum period and lactation), and for fueling infant and toddler growth (until two years of age). Failure to provide key nutrients during the first 1000 days of life can result in developmental shortfalls such as a lifelong deficit in brain function. To help optimize development and to fuel a healthy pregnancy, all essential nutrients should be included in the diet. This review distinguishes eight key nutrients and describes the unique role that each play during the first 1000 days of life, including carotenoids (lutein + zeaxanthin), choline, folate, iodine, iron, the omega-3 fatty acids, and vitamin D. Other nutrients, including B vitamins, magnesium, vitamin A and other trace minerals, are discussed, as they relate to topics such as maternal, infant and toddler nutrient deficiency and supplementation. 

The upcoming 2020–2025 Dietary Guidelines for Americans (DGA) will make specific recommendations for children, and it is important to provide accurate scientific information to support health influencers, such as dietitians and other health professionals, in the field of nutrition. Therefore, the purpose of this review is to 1) summarize the available scientific evidence regarding physiological and nutritional requirements during the first 1000 days of early life; 2) describe scientific data on the benefits of dietary nutrition supplements; and 3) provide professionals with a nutritional guidance document on pregnancy through early childhood. 

## 2. Materials and Methods 

### 2.1. Search Methods 

**Figure 1:**
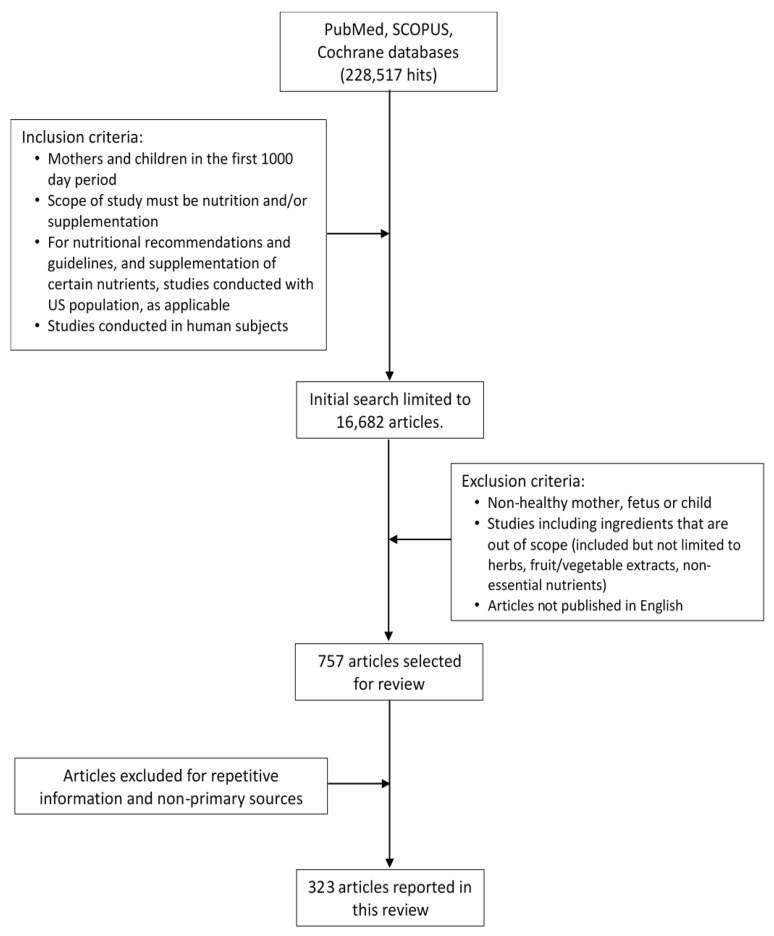
Overview of Literature Search Strategy and Results.

A narrative literature review was conducted in February of 2019 with a team of nutrition research scientists (Figure 1). An initial, comprehensive search for all research was conducted in PubMed, Scopus, and Cochrane databases to understand the current research available surrounding intake, deficiency and supplements of key nutrients during the first 1000 days life period. Due to the large scope of topics covered in this review, a supplement has been included to provide an example of the search terms used (Figure S1). The search strategy did not place any limits on date, however, to ensure this review provides up-to-date information, preference is given to data from the last ten years (since 2009). Further publications were gathered from a manual search of the reference lists of articles and reviews, and from discussion with field experts. A thorough literature search revealed that information was not always available regarding nutrition physiology, deficiency and supplementation of nutrients. Information on nutrients was included in this review only when evidence was available. 

### 2.2. Selected Literature 

The information in this review can be categorized as pertaining to either maternal health or fetal and child health. The literature for maternal health included only those titles discussing pregnant mothers, mothers who are less than two years postnatal, and mothers who are breastfeeding. Articles discussing the period pre-conception and women beyond two years postnatal were not included. The included literature for fetal and child health included titles discussing healthy embryonic and fetal development, neonates born at term, and healthy infants and toddlers up to two years old. 

The purpose of this review is to report outcomes related to usual and expected pregnancy and development, therefore disease states and unusual growth and development were out of scope. For mothers, articles discussing chronic illness, pregnancy complications, or poor birth outcomes, maternal hospitalization, maternal gastric bypass, and maternal, enteral or parenteral nutrition, were not included. For the child, articles discussing abnormal fetal development, birth defects, premature infant development, abnormal child development, child hospitalization, and child enteral or parenteral nutrition, were not included. 

## 3. Why Nutrition Matters: Maternal Physiological Changes and the Role of Nutrition

The physiological changes that occur during pregnancy are unique in the life of women. These changes are normal adaptations that occur to nurture the developing fetus and to prepare the mother for a healthy labor and delivery [2]. These changes begin immediately after conception and affect organ systems including the cardiovascular, endocrine, gastrointestinal, hematological, respiratory, and skeletal system [3]. For women who experience a normal and healthy pregnancy, these changes typically resolve after birth with minimal residual effects. Table 1 provides an overview of the major physiological changes that occur during pregnancy.

Physiological changes during pregnancy result in changing nutritional needs. During the first 2–8 weeks of pregnancy, foundational growth of the fetus occurs, and the nutrition status of the mother impacts early embryonic development, organogenesis, and neural development. During the second and third trimesters, fetal nutrients accumulate to be used after birth; therefore, it is critical to have an adequate supply of all essential nutrients. While it is important to recognize that all essential nutrients are required to support a healthy pregnancy and early childhood development, due to the scope of this review, we will focus on the role of eight key nutrients, including carotenoids (lutein + zeaxanthin), choline, folate, iodine, iron, omega-3 fatty acids and vitamin D.

**Key Takeaways:**

- The first 1000 days of life represents the time from pregnancy through the child’s second birthday.
- Mothers undergo major physiological changes to maintain pregnancy and prepare for a healthy labor and delivery; these changes begin after conception and affect all organ systems’ development, but especially the fetal cardiovascular, endocrine, gastrointestinal, hematological, respiratory and skeletal systems.
- Optimal nutrition status during pregnancy is critical, as it impacts early embryonic development, organogenesis and neural development.
- Nutrients such as the carotenoids (lutein + zeaxanthin), choline, folate, iodine, iron, omega-3 fatty acids and vitamin D play critical roles during fetal development.

### 3.1. Optimal Nutrition to Help Sustain A Healthy Pregnancy and Critical Periods of Development

**Figure 2:**
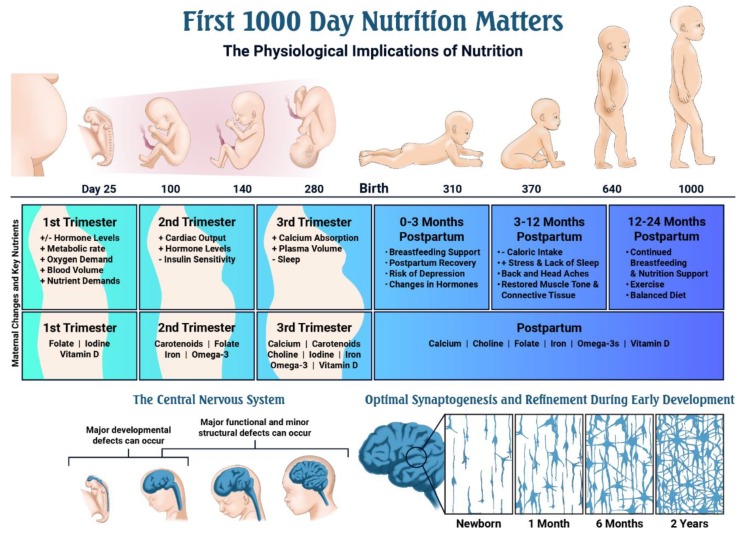
Why Nutrition Matters: a timeline of critical events during pregnancy and early development, and the role of nutrition.

Pregnancy places unique demands on a woman’s body with additional energy and increased intake of nutrients required to help support optimal fetal development [5] (Table 2). The body can be extremely sensitive to damage caused by internal and external harmful exposures (alcohol, medications, environmental toxins), and these exposures can trigger major or minor functional and structural fetal defects (Figure 2). Similarly, the body is sensitive to diet and nutrition. For example, in the presence of a healthy diet that delivers adequate amounts of key macro and micronutrients, fetal growth and development typically thrive [1]. 

Adequate nutrition is especially critical for normal central nervous system development. Neurological development is extremely rapid during the first 1000 days of life, with changes occurring from post conception (day 18) up until age two. Nerve cells proliferate at an extremely rapid pace, especially during early fetal development [6]. This growth culminates in a network of billions of neurons and trillions of neural connections by the time of birth [6]. During fetal and early childhood development, the prefrontal cortex, hippocampus, and sensory systems undergo tremendous development that will not be able to occur later in life [1] (Figure 2). 

Regarding energy requirements during pregnancy, extra calories should come from nutrient-dense foods to support a healthy pregnancy weight gain [5]. To help meet nutrient requirements, expectant mothers should consume foods that provide carotenoids (lutein + zeaxanthin), choline, folate, iodine, iron, omega-3 fatty acids, and vitamin D. In this section, we describe the role of each nutrient during pregnancy, as well as each nutrient’s role during critical periods of growth and development, where information is available. 

#### 3.1.1. Carotenoids

The carotenoids lutein and zeaxanthin play important roles during the development of the infant eye and brain. Lutein and zeaxanthin have been found to accumulate in the eye of fetuses as early as 17 to 22 weeks of gestation [13,14,15]. Therefore, the mother must have adequate lutein consumption to supply her own needs along with the needs of her unborn child. Studies have shown that lutein is not only present and often the predominant carotenoid in the mother’s bloodstream during pregnancy, but that lutein concentration typically increases during pregnancy, while levels of other carotenoids remain fairly constant [16,17].

Lutein levels in both cord blood and maternal plasma peak during the third trimester, a period of active retinal and neural development [18,19]. Lutein has also been found to be present at higher amounts in cord blood compared to other carotenoids [16,20,21]. Additionally, of the carotenoids present in the placenta, lutein and zeaxanthin were the most prevalent and levels were significantly correlated with levels in maternal serum and infant cord blood [22]. Placenta and umbilical cord blood rely on the mother’s dietary intake, thereby reemphasizing the importance of maternal nutrition during pregnancy.

Lutein and zeaxanthin have established roles as antioxidants and visual filters. Their presence in the eye may serve as a protective factor against oxidative damage during early development due to the high metabolic activity of this tissue, abundance of long-chain polyunsaturated fatty acids, and vascularity. Beyond protection, these carotenoids also support neuronal development via stabilizing microtubules [23], enhancing gap junction communication [24], improving vasculature [25], and stabilizing and modifying the permeability of membranes [26]. 

#### 3.1.2. Choline

Choline, a precursor to acetylcholine, is an essential nutrient that aids in cell membrane signaling and transporting lipids via lipoproteins [27]. Choline is also required to synthesize phospholipids including phosphatidylcholine and sphingomyelin, both of which are essential components of cell membranes. During pregnancy, the requirements for choline increase because of elevated maternal demand and the rapid division of fetal cells [28]. Choline can also influence stem cell proliferation and choline insufficiency can promote cellular apoptosis. As a result of insufficiency, brain and spinal cord structure may be altered, increasing the risk for neural tube defects [27].

Beyond its ability to synthesize neurotransmitters and molecules necessary for normal functioning of the human body, choline also plays a vital role in cognitive development. During the later stages of pregnancy, the hippocampus (the memory center of the brain) develops and continues to develop after birth and up until four years of age. A lack of choline in the maternal diet during critical periods of fetal development may cause lifelong changes in a child’s brain structure and function, including the hippocampus. New evidence also suggests that sufficient maternal choline intake during pregnancy and lactation can have long-lasting beneficial neurocognitive effects on the offspring [29].

Furthermore, cross-sectional data also reveal that consumption of choline from foods and beverages is not optimal. Data from What We Eat in America, NHANES 2015–2016, demonstrate that women of childbearing age, 20 years and over, consume about 287 mg per day of choline from foods and beverages, which is considerably below the Adequate Intake (AI) recommendation of 425 mg per day for non-pregnant women and even further below the recommendation for pregnant women (450 mg per day). Ensuring that women of childbearing age receive optimal amounts of choline in their diet should be made a public health priority, to decrease risk for neural tube defects and to foster the healthy growth and development of young children [30]. 

#### 3.1.3. Folate

Folate is a B-vitamin important for both fetal and maternal health, functioning as a coenzyme critical for DNA synthesis and amino acid metabolism. Folate is a generic term that includes both the naturally occurring forms of the vitamin (from food) or folic acid, a form commonly found in dietary supplements and fortified foods. For women of childbearing age, folate is critical to normal neural tube development (the area from which the brain and spinal cord form) in the fetus within 28 days of conception. One of the other major functions of folate is that it provides single carbon units for the synthesis of nitrogenous bases (purine and pyrimidine) and amino acid metabolism, making folate essential for DNA synthesis [31]. This metabolic pathway is also important for erythropoiesis, which is rapidly surging during pregnancy to help increase the mother’s blood volume in preparation for the fetus.

#### 3.1.4. Iodine

Iodine is a micronutrient that works in tandem with the thyroid gland. The thyroid gland uses iodine from food to make two thyroid hormones including thyroxine (T4) and triiodothyronine (T3). During pregnancy, iodine requirements are increased by ≥ 50% due to increases in maternal thyroid hormone production necessary to supply to the fetus, which does not have a fully functional thyroid gland until 20 weeks gestation [1,32]. In the fetus, iodine is important for normal brain and nervous system development [33]. 

#### 3.1.5. Iron

Iron is a trace mineral that is required for fetal growth and development, because it serves as a cofactor for enzymes involved in oxidation–reduction reactions, which occur in cellular metabolism. Iron is also a major component of hemoglobin, the protein that allows red blood cells to carry oxygen throughout the body. The neonatal brain is in an active metabolic state consuming about 60% of total body oxygen (in comparison, the adult brain consumes about 20% of total body oxygen); therefore, pregnant women have high demands for iron. Pregnancy also requires a large expansion in blood volume to meet the demands of the growing fetus. 

A prospective cohort study compared nutrient intake levels during pregnancy to recommended intake levels and found that none of the pregnant women (*n* = 200) achieved the recommendation for dietary iron [34]. Meeting the iron recommendation of 27 mg per day is especially important during the last trimester of pregnancy, since the fetus accumulates iron for use during early life. 

Research has shown that if pregnant women are iron deficient, and, consequently, iron is not available to the infant in the first six months of life, there can be lifelong irreversible neurological effects [35]. Furthermore, women who meet iron requirements during pregnancy may provide an advantageous impact on cognitive development in their children [36,37]. In the first year of life, iron continues to play a vital role in neurodevelopment. During this time, the brain experiences a considerable transformation, becoming a complex organ. Several neurodevelopmental processes occur, including synaptogenesis, the organization of neurotransmitter systems, and the onset of myelination, especially within the hippocampus, visual and auditory systems. Iron is also associated with critical cellular processes in the brain, including the maintenance of neural cell energy and neurotransmitter homeostasis. Collectively, because the brain continues to develop during infancy and early childhood, iron may have an influence on cognitive ability and behavior [36]. 

#### 3.1.6. Omega-3 Fatty Acids

Omega-3 fatty acids include alpha-linolenic acid (ALA, 18:3n3), eicosapentaenoic acid (EPA, 20:5n3, docosapentaenoic acid (DPAn3, 22:5n3) and docosahexaenoic acid (DHA, 22:6n3) [38]. All of these are significant dietary components, however, EPA and DHA will be the primary focus of our discussion, with DHA being the principal omega-3 found in mammalian tissues. ALA, an essential omega-3 fatty acid, is enzymatically converted in vivo to EPA (which can subsequently be converted to DHA). However, due to enzymatic competitive inhibition, this process is inefficient. Therefore, direct consumption of DHA is optimal to achieve ideal circulating levels [39]. 

Omega-3 fatty acids, DHA in particular, are important for supporting a healthy pregnancy. There is an active transport of DHA and other polyunsaturated lipids across the placenta [40] to support the high demands for fetal growth, especially during the last trimester [41,42]. Crawford, et al., have described the process where there is an increased concentration of DHA going from mothers’ bloodstream to the fetal bloodstream to the fetal brain as “biomagnification”. This process is suggested to provide for optimal fetal brain development and rapid accumulation of a high concentration of nervous system DHA [43]. 

Mothers who have healthy intakes of DHA give birth to infants with more bloodstream DHA and better visual function as measured by visual evoked potentials [44]. The continued intake of EPA and DHA are also important for the maintenance of the mother’s cardiovascular health, as they mitigate several risk factors for disease, including lowering triglycerides and LDL, raising HDL and modulating blood pressure, heart rate and arterial compliance [45]. The level of omega-3 fatty acids in the mother’s bloodstream during pregnancy has been shown to correlate with insulin levels [46] and adiposity [47]. Newer lines of inquiry have indicated that prenatal DHA during the second half of pregnancy alters the infant epigenome (gene activity changes that do not affect the DNA sequence) and can alter developmental programming [48]. 

#### 3.1.7. Vitamin D

During pregnancy, vitamin D plays a vital role in fetal growth and development by supporting the skeletal system, and the formation of tooth enamel, and by aiding in calcium regulation [49]. There is also some emerging evidence to support a role for vitamin D in fetal immune development and function [49]. During pregnancy, maternal calcium is mobilized, and subsequent utilization increases to meet the demands of fetal bone mineralization. As a result, several physiological adaptations take place, including increased serum calcitriol, vitamin D binding protein, placental vitamin D receptor (VDR) and renal and placental CYP27B1 (the enzyme that produces the bioactive form of vitamin D) to maintain normal serum levels of 25(OH)D and calcium. Maternal 25(OH)D crosses the placenta and is the main form of vitamin D for the fetus. Additionally, vitamin D escalates calcium absorption and placental calcium transport during pregnancy, while also regulating immune system function and modulating inflammation. All these effects indicate how important vitamin D is during gestation [50]. Several observational studies show a relationship between inadequate serum 25(OH)D in pregnant women and adverse neonatal and pregnancy outcomes including preeclampsia, small for gestational age (SGA), preterm birth and gestational diabetes mellitus [51].

**Key Takeaways** **:**

- Pregnancy is a period of increased nutrient demands, when optimal nutrition is critical for maturing, proliferating, and differentiating cells throughout the fetus.
- Carotenoids play a key role in brain, eye and nervous system development.
- Choline fuels cell growth and proliferation, as well as nervous and cognitive system development.
- Adequate intakes of folate prior to and during pregnancy may help to prevent neural tube defects. Folate also plays a key role in DNA synthesis and amino acid metabolism.
- Iodine helps produce thyroid hormones, that are transferred to the fetus early in life.
- Iron is a major component of hemoglobin, a protein that allows red bloods cells to transport oxygen throughout the body.
- Omega-3 fatty acids are crucial for the development of the nervous system and eye, and overall fetal growth.
- Vitamin D supports the skeletal system, helps to regulate calcium levels by increasing calcium absorption, and may negate adverse pregnancy outcomes including preeclampsia, SGA, preterm birth and gestational diabetes mellitus.
- Ensuring that women of childbearing age receive optimal nutrition should be a priority for health professionals.

### 3.2. The Postpartum Period: Feeding Baby

Scientific organizations including the American Academy of Pediatrics (AAP) and the Academy of Nutrition and Dietetics (AND) recommend exclusive breastfeeding for six months, with continuation for one year or more, as desired by the mother [52,53]. A World Health Organization review along with an opinion paper published by the European Food Safety Authority (EFSA) described that exclusive breast feeding by well-nourished mothers for six months can meet the needs of most healthy infants for energy, protein, and for most vitamins and minerals, with the exception of vitamin K and vitamin D, both of which can be addressed by supplementation [54,55].

Lactating women require nutrients in increased amounts in comparison to non-pregnant women, including vitamins A, E, B6, B12, choline, folate, iodine, lutein and zeaxanthin, zinc, omega-3 fatty acids, as well as increased amounts of fiber and protein (Table 2). Nutrients that are low in breast milk include zinc, iron, and vitamin D. Several scientific organizations recommend supplemental vitamin D to infants, and especially to exclusively breastfed infants. Infants receive most of the required amount of vitamin D from sun exposure, and the rest from formula or breast milk [53,56]. For breastfeeding mothers, only minimal amounts of maternal serum 25(OH)D are transferred to human breast milk; therefore, to provide sufficient vitamin D content in breast milk for the infant, the vitamin D intake of the mother during lactation has to be much higher compared to the intake during pregnancy. Overall, mothers who choose to breastfeed can consider micronutrient supplementation [1].

While breastfeeding is considered the gold standard for feeding infants, it is not always possible for all mothers to achieve. Potential barriers to breastfeeding include overall discomfort, improper latching, lack of knowledge or uncertainty about breastfeeding, and the stress of returning to work [57]. Breast milk and formula provide infants with water, carbohydrates, human milk oligosaccharides, essential fatty acids, proteins, carotenoids, and vitamins and minerals [58]. Human milk contains nutrients, growth factors and cells important for brain development that formula lacks, however, formula contains vitamin D, iron, and omega-3s that may be insufficient in breast milk, especially if the mother is deficient [1]. It is important for families to consult with health professionals to develop a feeding plan that gives the mother and baby the best chance for health and healthy development. 

**Key Takeaways:**

- AAP and AND recommend exclusive breastfeeding for 6 months, with continuation for ≥1 year, or as desired by mother.
- Health professionals should be aware of the differences between breast milk and formula to help families make healthy feeding plans for their infants.
- Lactating mothers have increased nutrient needs, and they may be deficient in iron, zinc, and vitamin D.
- While breast milk is the gold standard for feeding, breastfeeding or formula feeding can be the primary source of nutrition for the growing child.

## 4. Nutrition for the Growing Child, 0–24 Months

Infants change dramatically in the first 24 months of life and each child can vary considerably in terms of their growth, development, and feeding patterns (Figure 2). Immediately after birth, newborns lose about 5%–10% body weight, until about two weeks of age, when they have established good feeding patterns, begin to gain weight, and grow. From birth until two years of age, infants and toddlers have extremely high metabolic rates and calorie needs. Newborns, for example, require about 50 calories per pound daily to support rapid growth and a high basal metabolic rate. After two to three months of age, calorie needs decrease to about 40 calories per pound and remain at this level until the age of three [59]. 

Complementary foods are defined as solids or liquids other than breast milk or infant formula. The introduction of complementary foods represents a period when breast milk or formula alone is no longer enough to meet the nutritional demands of infants, usually at four to six months of age [54]. During this time, breast milk or formula should be continued, but infants should be offered complementary foods with a variety of flavors and textures; this is necessary for both nutritional and developmental reasons [54]. In terms of specific nutrients required by growing children, Table 3 provides the Dietary Reference Intakes (DRI) for key nutrients required for healthy growth and development (by stage). Nutrients such as fat, including the essential fatty acids, linoleic and alpha-linolenic acids, are an important determinant of energy supply throughout the first year of life and should be well supplied in the diet. Even though a DRI has not been established for carotenoids such as lutein and zeaxanthin, there is a growing body of evidence that these nutrients are important for the development of the visual and neural systems and have a positive impact on health outcomes [20,25].

At six months of age, requirements for other nutrients, such as iron and zinc, increase dramatically (Table 3). By this time, the infant’s internal stores are depleted and the need for iron and zinc increases, as the physiological requirement per kg body weight becomes greater than later in life [54]. Due to the quick growth and metabolic rate during this stage, the nutrient density of foods offered in the diet needs to be high. Dairy products, pulses, and leafy green vegetables should be included in the diet when possible, since they are key sources of protein, calcium, and vitamin D, which are required to support the growth of healthy bones and prevent rickets [54,55]. Furthermore, foods that provide adequate levels of vitamin A, C, B6, B12, and folate are particularly important to include in toddlers’ diets, as they can help to prevent major nutrient-related deficiencies, enhance non-heme iron absorption, and foster healthy growth and development [54]. A study of US toddlers between 18 and 36 months indicates a very low DHA intake of about 20 mg per day which is consistent with NHANES reports of intakes of 20 mg/day in children under 6 years of age [60]. Correcting this deficiency led to an improvement in their respiratory health [60]. 

**Key Takeaways:**

- By 6 months of age, infant iron and zinc stores are depleted, therefore foods that contain these nutrients should be preferentially offered.
- From 6 months onward, growth and development continue to be rapid, and nutrients such as protein, calcium and vitamin D are required for accretion of skeletal mass and to help prevent nutritional rickets.
- Omega-3 fatty acids, specifically DHA, are required for continued brain and eye development.
- Carotenoids, such as lutein and zeaxanthin, continue to play key roles in eye and neural development.

## 5. Nutrient Deficiency During the First 1000 Days

### 5.1. Maternal and Fetal Nutrient Deficiency

Optimal nutrient intake during the first 1000 days is critical for the wellbeing of both mother and baby. Nutrient deficiencies, even mild ones, may result in detrimental effects to embryonic development that may compound over time, leaving a lasting effect on the health of the infant for a lifetime [61,62]. Maternal health can be similarly impacted by nutrient deficiencies during this time, when a mother’s ability to nurture, feed, and care for herself and her child is dependent on her health [31,63,64,65]. The goal of this section is to discuss nutrient deficiency during the first 1000 day period and its impact on maternal and child health. 

#### 5.1.1. B-Vitamins

The rates of deficiency for vitamins B6 and B12 are not well documented, however the role of vitamin B9 (folate) in fetal development continues to be one the most well-studied of all nutrients during pregnancy [9,66,67,68,69,70]. An analysis of NHANES 2001–2014 data of pregnant women found mean intakes of vitamin B6, folate, and vitamin B12 from food averaged near the EAR, and intake from food and supplements averaged at least double the EAR [71]. 

Recent data suggest the rate of folate deficiency in pregnancy in the United States is as low as 0.5%, since mandatory grain fortification started in 1998 [72], dramatically reducing the number of birth defects, especially spina bifida [66]. Bailey, et al., (2019) found folate intakes from both food and supplements were at risk of inadequate intake, with 16.4% of pregnant women below the EAR, however, at the same time, 33.4% of pregnant women had intakes above the Tolerable Upper Intake Level (UL) [71]. This wide disparity in intakes suggests a folate source that is not consistent across the population, potentially from inconsistent prenatal vitamin use by expectant mothers [71].

Much of the research that exists for B vitamin deficiency during pregnancy is focused on vitamins B6, B9 (folate), and B12 and their role in early fetal development [70]. Recent research suggests that maternal vitamin B12 deficiency may result in neural tube defects, as well as other congenital malformations and an increased risk of first trimester miscarriage [70]. A maternal deficiency in vitamins B6 and B12 negatively impacts cell division and early development of the embryo, even before implantation, which may also result in congenital malformations and increased miscarriage risk [70]. For the mother, vitamin B12 deficiency can cause mental slowness, memory problems, and numbness or tingling in the extremities [73].

#### 5.1.2. Calcium

During gestation, the average fetus requires approximately 30 grams of calcium [74,75]. Calcium is critical for fetal bone development as well as maintenance of the maternal skeleton and healthy smooth muscle contractions [74]. Most fetal calcium transfer occurs during the third trimester; approximately 80% of the calcium in the fetal skeleton is transferred from the mother during this time [74,75]. To maintain the high calcium needs and partially spare maternal bone content, the efficiency of intestinal calcium absorption more than doubles during pregnancy as early as week 12 [74]; enhanced intestinal calcium absorption has been shown to increase 24 h urine calcium excretion. However, during periods of fasting, urine calcium levels are low [76], indicating a state of absorptive hypercalciuria [74]. Prevalence of calcium deficiency during pregnancy in the Western world is low, but in developing countries, or populations with historically low dietary calcium intake, the World Health Organization recommends daily elemental calcium supplementation of 1.5–2 grams [77]. 

#### 5.1.3. Carotenoids

The prevalence of carotenoid deficiency, including from lutein and zeaxanthin, is unknown. However, some studies have revealed worse health outcomes and increased risk of certain conditions with reduced maternal blood lutein concentrations. A study conducted by Cohen, et al., revealed the risk of preeclampsia decreases with increased plasma lutein concentrations [78]. As pregnant mothers with preeclampsia have been found to have an increase in oxidative stress and lower antioxidant status, lutein’s ability to act as an antioxidant may help reduce the risk of this condition. Additionally, high maternal plasma concentrations of carotenoids during pregnancy decreased the risk of giving birth to SGA babies [79]. While these studies suggest a link between maternal lutein and zeaxanthin status and reduced risk of preeclampsia and SGA babies, a better understanding of these associations is needed, as one cannot rule out confounding variables such as physical activity, nutrition, or demographic characteristics.

#### 5.1.4. Choline

Severe choline deficiency is rare during pregnancy in the United States [80], however, 95% of all pregnant women do not meet the recommended AI of 450 mg per day for choline [81]. The intake for choline from food and beverages during pregnancy is less than 350 mg per day [82]. An analysis of pregnant women in NHANES 2009–2014 found a usual intake of 319 ± 9.89 mg per day of choline, with only 8.51% ± 2.89% of them having an intake of choline greater than the AI [29,83]. This intake value was similar to the mean intake of choline from foods and supplements by pregnant women found recently by Bailey, et al., (2019) in NHANES data from 2001–2014, of 322 mg (SE = 10.6 mg) per day, with the vast majority of the choline intake coming from the food they were consuming [71].

Choline intake during pregnancy has been correlated with many areas of cognitive development in the fetus and newborn. Pregnant mothers with adequate choline status or intakes at or near the AI for choline are shown to give birth to infants who have a significantly reduced risk of behavioral problems at 12 months [84], and a higher cognitive development score at 18 months [85] compared to women with poor choline status and intakes. No significant relationship was found between maternal choline intake and the risk of neural tube defects [86,87], however, women who have poor folate status during pregnancy are at increased risk for choline deficiency [88,89]. 

#### 5.1.5. Magnesium

The prevalence of magnesium deficiency during pregnancy is unknown, however, many adults in the United States do not meet the Recommended Dietary Allowance (RDA) [1,90,91] and low plasma magnesium is common [92]. Women in the United States aged 20 years and older typically consume 277 mg per day of magnesium [30], which is significantly less than the 310 mg per day required for adult females and the 350 mg per day required for pregnancy. An analysis of NHANES 2001–2014 data of pregnant women found a mean intake of magnesium of 294 mg (SE = 6.4 mg) per day from food and a slightly higher intake of 314 mg (SE = 7.2 mg) per day from food and supplements [71]. Even with supplementation, 47.5% of pregnant women had magnesium intakes below the EAR [71].

Deficiency in magnesium during pregnancy is associated with preeclampsia and pre-term delivery in the mother [93] and poor organ growth in the first trimester of the fetus, especially the vascular system [70,94]. Overall, this may result in intrauterine growth restriction (IUGR) and low birth weight (LBW) in the newborn [93]. 

#### 5.1.6. Omega-3 Fatty Acids

The prevalence of EPA and DHA deficiency during pregnancy is not adequately researched. A recent worldwide survey of plasma and erythrocyte EPA and DHA content reported a widespread dietary inadequacy, particularly in Western societies [95]. An analysis of NHANES 2003–2014 dietary intake and supplement data found pregnant women had a mean intake of EPA of 33.1 mg (SE = 4.1 mg) and DHA of 67.5 mg (SE = 11.8 mg) per day, and that 7.3% of pregnant women reported taking an EPA or DHA-containing supplement [96]. An analysis by Zhang, et al., (2018) looked at the 2001–2014 NHANES data and found 94.48% of pregnant women were not meeting the 2015–2020 Dietary Guidelines for American’s (DGA) of 250 mg of EPA and DHA per day [97]. Women who give birth to twins, triplets, or multiples exhibit lower bloodstream DHA, and the infants appear to have divided the available omega-3 fatty acid from the mother between them [98].

#### 5.1.7. Trace Minerals

Maternal deficiencies in trace minerals during pregnancy primarily impact the fetus, and many of the most detrimental effects can be seen during the first trimester, before the pregnancy is known. Trace minerals have a role in early fetal organ system development as important players in cell growth and differentiation [70,73,99,100]. This effect is seen most prominently in growth of the vascular system, kidneys and pancreas, and deficiency may predispose the child to cardiometabolic diseases as an adult [70].

##### Zinc

The global prevalence of zinc deficiency during pregnancy is estimated to be 82%, with a significantly larger portion of these cases being in poorer, underserved families [99]. A meta-analysis of data from developed countries found a median intake value of 11.3 mg (interquartile range of 10.4–12.3 mg) of zinc per day in the US and Canada [101]. This suggests the prevalence of zinc deficiency may be low in American women during pregnancy, although further research is warranted. Zinc deficiency slows the development of the placenta, which can then impede organogenesis [99]. Zinc deficiency may also increase the risk of preterm labor, spontaneous abortion, and LBW, although its mechanisms are unclear [102].

##### Copper

Copper deficiency is uncommon both in the United States and globally [103], and little is known about the prevalence of copper deficiency during pregnancy. Based on animal studies, there is evidence that copper deficiency can affect early embryonic development pre- and post-implantation, by affecting the development of connective and nervous tissue [100]. 

##### Iodine

In the United States, which has historically been iodine sufficient, 56.9% of pregnant women had iodine intakes that were significantly less than the RDA [104,105]. A recent analysis of the 2009–2010 NHANES survey found that the median urinary iodine concentration in pregnant women was significantly lower than previous years [106], potentially suggesting that the number of iodine-sufficient pregnancies is decreasing. This same study found no significant correlation between urinary iodine and salt intake (iodized and non-iodized salt combined) but did find a significant correlation with racial/ethnic minority status [106]. Maternal iodine deficiency can cause mental slowness and tiredness in the mother and can slow or influence nervous system growth during the first and second trimesters of fetal development [70,107]. Severe maternal iodine deficiencies can cause significant fetal developmental delays and result in an infant born with cretinism [107].

##### Iron

Iron deficiency and the resulting iron deficiency anemia (IDA) is very common in the United States. An estimated 18% of pregnant women in the United States have some form of iron deficiency [108], commonly a nutritional iron deficiency [73], caused by the increased iron demands of pregnancy [70,73]. Many women with mild iron deficiency are asymptomatic, while many others have symptoms like lethargy, weakness, irritability, and poor work tolerance [73]. Maternal iron deficiency can result in a number of potentially serious consequences during pregnancy, including a smaller placental size [70] and slowed organogenesis in the first trimester [73]. This impacts fetal growth and can increase the risk of chronic fetal hypoxia, low iron stores in the newborn, poor birth outcomes, poor cognitive development, and cardiometabolic disease for the child later in life [70,73,109].

#### 5.1.8. Vitamin A

The prevalence of vitamin A deficiency in the United States and other developed countries is low [110]. A 2001–2014 analysis of NHANES data of pregnant women found a mean intake of vitamin A from food of 696 μg (SE = 27 μg) per day and a mean intake of 1283 μg (SE=54 μg) per day of vitamin A from food and supplements combined. Even with supplementation, 15.5% of these pregnant women had vitamin A intakes below the EAR [71]. 

The first 1000-day period is a critical time to ensure adequate intake of vitamin A in pregnant women in high-risk geographic areas for deficiency [110]. The requirement for vitamin A to the fetus is highest during the third trimester [111], and both the mother and child may be affected by deficiency. Maternal deficiency can result in night blindness and is accompanied by an increased risk of mortality [112]. Fetal vitamin A deficiency increases the risk of preterm birth and mortality [111,112].

#### 5.1.9. Vitamin D

Currently, there is no universal definition of vitamin D deficiency [51], and the standards for which adequacy levels were established only considered the levels necessary to maintain bone health [12]. In pregnant women, deficiency prevalence (<50 nmol/L) is estimated to be between 40%–60% in the U.S. [113,114], and as high as 54% globally [115]. Some studies based in the United States estimate this number to be around 66% [116] and find the prevalence of deficiency to be six times higher in African American women, compared to Caucasian women [114]. Bailey et al., (2019) found mean vitamin D intake of 5.5 μg (SE = 0.2 μg) per day from food, and a mean vitamin D intake of 11.3 μg (SE = 0.4 μg) per day from food and supplements using NHANES 2001–2014 data [71]. Nearly half of these women had vitamin D intakes below the EAR, even with supplementation [71], highlighting the need for increased attention to vitamin D supplementation in pregnant women. In pregnancy, vitamin D deficiency increases the risk of placental insufficiency and preeclampsia, is associated with increased risk of bacterial vaginosis, and increases the risk of developing gestational type 2 diabetes (T2D) by increasing glucose intolerance [113,117,118].

**Key Takeaways:**

- During the gestational period, expectant mothers can become deficient in various vitamins and minerals, due to the physiological demands of pregnancy.
- Vitamin and mineral deficiencies during pregnancy include folate (especially during the first trimester), vitamin D, iodine, iron, magnesium, choline, carotenoids, and omega-3 fatty acids.
- Deficiency of fat-soluble vitamins including A and D, put the expectant mother at risk for night blindness and the increased risk for adverse pregnancy outcomes, such as preeclampsia or SGA.
- There is a wide range of intakes for many nutrients across the U.S. population, including iron, vitamin B6, folate, vitamin B12, lutein, and zeaxanthin, suggesting pregnant women can benefit from the guidance of health professionals to promote optimal intakes and prevent nutrient excesses.

### 5.2. Maternal Postnatal Deficiency

Women suffer from a wide range of issues in the postpartum period that may be influenced by nutritional status, including excess bleeding, urinary incontinence, hair loss, depression, headaches, and feelings of stress and fatigue [119,120]. Nutritional status related to chronic diseases in the postpartum period, including diabetes, metabolic disorders, obesity, thyroid disease, renal disorders and mood disorders, are also of relevant concern [120]. The role of nutrition in maternal postpartum health has not been well-researched, and there is little attention given to postpartum health, especially when compared to prenatal health [119,120,121].

### 5.3. Nutrient Deficiencies in Infants and Toddlers, 0–24 Months

#### 5.3.1. B-Vitamins

Vitamin B deficiencies are rare in infants [122]. Analyses from large dietary intake studies have found that most children aged 6–24 months are meeting recommendations for vitamins B6 [123,124], folate [124,125], and B12 [124] from food and supplements. 

Folate deficiency is rarely found in newborns, but deficiency does increase slightly with age. Some folate deficiency can be found in less than 10% of preschool-aged children [122]. Vitamin B12 deficiency is also rare in infants, although it is found more often in breastfed children [126]. It is usually caused by maternal deficiency during pregnancy [126] and exacerbated by inadequate B12 in breast milk [126], especially in vegetarian mothers [122]. Signs of B12 deficiency in breastfed infants include hypotonia, developmental delays, and failure to thrive [126]. 

#### 5.3.2. Carotenoids

The prevalence and impact of carotenoid deficiency, including lutein and zeaxanthin, on infants and toddlers has not been adequately documented. However, the ratio of lutein and zeaxanthin in the eye is linked to anatomical development after birth [127]. At birth, the infant eye is not fully developed, having distinct physiological differences from that of the adult eye [128]. Maturity of the retinal area does not occur until 4–7 years of age [129,130,131,132]. Due to increased metabolic activity during this time of development, the young retina is very susceptible to oxidative damage. Additionally, the clarity of the lens and the short optical distance between the lens and the retina may allow significant transmission of all wavelengths of visible light into the eye [133,134]. As lutein and zeaxanthin are concentrated in the area of the retina that is most immature [135], they protect the developing retina through a number of mechanisms, namely absorption of blue wavelengths of visible light [136,137], antioxidant capacity [138,139], anti-inflammatory properties [140], and neuroprotective activity [141]. Beyond protection, they can also support transmission and processing of visual information [133,134]. These data suggest lutein and zeaxanthin have a key role in visual and neuronal development and a lack of these nutrients could have a detrimental effect on the growing infant.

#### 5.3.3. Choline

Little data exists on choline deficiency prevalence for infants and toddlers. An NHANES analysis of 2009–2012 data found children 6–24 months had mean choline intakes from food that met the AI [123]. An NHANES analysis of children 1–3 years old found males had mean choline intakes of 221 ± 7.6 mg per day and 61% had intakes above or equal to the AI, while female children ages 1–3 years had mean choline intakes of 205 ± 4.8 mg per day, and only 40% had intakes above or equal to the AI [29].

In the first six months of life, choline status is dependent on maternal choline status for breastfed infants only, as formula-fed infants receive choline from formula [142]. It is well-documented that the first months of life are critical for nervous system development, and choline status is linked to brain development during this time [82,142]. It is unclear what effect choline deficiency has on influencing infant brain development [27,82], but rat studies indicate choline deficiency may result in reduced capability for memory and learning [27,143]. 

#### 5.3.4. Magnesium

Little is known about the prevalence of magnesium deficiency during infancy and early childhood. A 2005–2012 NHANES analysis found that magnesium intakes from food increased by age in young children from 52.7 ± 1.2 mg per day in children ages 0–5.9 months to 172.1 ± 3.3 mg per day in children 12–24 months [144]. A study of food and supplement intake found 83% of the children less than 6 months had magnesium intakes above the AI, while 72% of the children ages 6–11.9 months had intakes above the AI [124].

Magnesium is linked to the parathyroid gland and bone remodeling, and deficiency can cause hypoparathyroidism, hypocalcemia, and poor bone growth in children [94]. This can be especially severe and result in lifelong issues in children under two years old, because of the rapid growth and development in this age group [94].

#### 5.3.5. Omega-3 Fatty Acids

The prevalence of omega-3 fatty acid deficiency in infants and toddlers in the United States is not well documented. Of the main omega-3 fatty acids, DRI exists only for ALA. A 2009–2012 NHANES analysis found that children 6–11.9 months had a mean intake of 0.65 ± 0.02 g of ALA per day, and children 12–23.9 months had a mean intake of 0.86 ± 0.02 g of ALA per day [123]. In the same analysis, 70% of both age groups met or exceeded the AI [123]. There are suggestions that DHA can aid in preventing some of the pathological conditions to which premature infants are prone to, such as retinopathy of prematurity [145]. Omega-3 fatty acid deficiency during infancy can result in childhood stunting, reduced birth rate and increased risk of infant mortality [146].

#### 5.3.6. Trace Minerals

Infants and toddlers require lesser amounts of trace minerals from their diet, but may be more prone to deficiency than adults, because of rapid growth and uneven intake [71,147,148].

##### Zinc

Globally, around one half million deaths are related to zinc deficiency, and many of these deaths occur in children under five years old [99]. Severe deficiency is rare in industrialized nations, but it is replaced with a widespread, persistent, mild zinc deficiency [99]. In children in the United States less than 12 months old, mean intake of zinc from food was found to be well above the AI and EAR [144]. In children 12–24 months old, a NHANES 2003–2012 analysis found the mean intake from food was 7.1 mg (95% CI: 6.9, 7.4) per day, and over 50% of children had usual intakes above the UL [149]. This aligns with other large dietary analysis studies that have found comparable results [123,124] and suggests zinc deficiency is not common in the United States.

Exclusively breastfed infants and toddlers with diets high in processed foods, and children who were born premature or had an LBW are at the highest risk for zinc deficiency [150,151]. Zinc deficiency in infants and toddlers affects cell types with high turnover rates, and a mild deficiency can result in alopecia, diarrhea, dermatitis and poor appetite [99]. A moderate zinc deficiency manifests as growth retardation and poor muscle mass, cognitive developmental delays, and altered immune functioning that includes poor wound healing, increased inflammation, and increased allergic sensitivity [102]. 

##### Copper

Copper deficiency is rare but is associated with malnutrition in infants and toddlers [147,152]. An NHANES analysis of 2009–2012 data found 99% of children ages 6–11.9 months had a mean daily food intake of copper that met or exceeded the AI [123]. In children 12–24 months old, the mean daily food intake met or exceeded the EAR, with 7% exceeding the UL [123]. Copper deficiency can occur when a child is recovering from diarrhea-related malnutrition, or when an infant is fed cow’s milk [147], and can lead to anemia, growth retardation, altered glucose metabolism, and an impaired immune system [153]. 

##### Iodine

While iodine has not been included in the USDA’s national nutrient databases, work is being done to use new analytical methods to measure the iodine content of foods that are expected to be contributors of iodine to the US diet [154]. Therefore, there are no reported intake values from large studies that can estimate national iodine intake. One recent study of 60 U.S. infants, ages 1–14 months, completed iodine analysis of foods consumed by the infants and found the estimated daily intake of iodine to be 89 μg per day [155].

Iodine status in infants is often linked to maternal iodine status, as a product of placental and breast milk transfer of iodine [70,107,156,157]. Severe iodine deficiency results in significant physical and mental developmental delays during childhood, but the most apparent effect is seen in infancy and toddlerhood, when neurocognitive development is rapid [158,159,160]. Mild iodine deficiency and its relationship to childhood disorders is difficult to measure, but there is some evidence to indicate that even a mild deficiency can cause reduced mental capacity, poor memory, and delayed cognitive development related to thyroid functioning in toddlers [156] and older children [161]. 

##### Iron

Little data exists on iron deficiency rates in infants under 12 months in the United States. An analysis of food and supplement intake data found that children ages 0–5.9 months had a mean intake of 6.2 ± 0.2 mg iron per day, with 100% meeting the AI. Children ages 6–11.9 months had mean intakes of 13 ± 0.2 mg iron per day with 18% not meeting the EAR [124]. 

The rate of deficiency in children 12–23 months old is 8% [162], and the rate of IDA in children 12–35 months is about 4% in the United States [163], making iron deficiency a preventable cause of disease in this age group. Bailey, et al., (2018) found children ages 12–24 months had a mean daily intake of iron from food and supplements that met or exceeded the EAR, but 7% of these children did not meet the EAR [124]. Infants and young children at the highest risk for IDA have an LBW, are from low- to middle-income areas, and consume high quantities of cow’s milk [164]. Iron deficiency in young children can result in impaired neurological and behavioral development, which is sometimes irreversible [163].

#### 5.3.7. Vitamin A

The prevalence of vitamin A deficiency in the United States and other developed countries is low [110]. In a variety of recent NHANES analysis studies that looked at nutrient intakes in this age group, children ages 0–23.9 months were found to have mean food intakes of vitamin A near or in excess of the UL, ranging from 537.8 ± 7.4 to 675.4 ± 12.4 μg RAE per day [125,144,165]. A similar result was found for vitamin A intakes including foods, beverages and supplements [124]. Vitamin A deficiency is a leading cause of preventable blindness in children, and significantly increases the risk of childhood mortality [110].

#### 5.3.8. Vitamin D

The prevalence of vitamin D deficiency in infants and toddlers is estimated to be between 40%–50% in the United States [166,167]. Recent, large population-based dietary analyses have found about three quarters of children ages 6–23.9 months do not meet the AI (children less than 12 months) or the EAR (children 12–23.9 months) for vitamin D [123,125,144]. Similar vitamin D levels were found in another study measuring intake from food, beverages and supplements [124]. 

A recent meta-analysis of observational studies showed an association between vitamin D deficiency and decreased head circumference-for-age at 1 year, but no relationship between deficiency and infant length at 9 months, or weight-for-age at 1 year [51]. The same meta-analysis showed the data is inconclusive for a relationship between vitamin D deficiency and newborn length, length-for-age at 1 year, risk of SGA, newborn head circumference, and onset of type 1 diabetes (T1D) because of inconsistencies between study methods [51].

**Key Takeaways:**

- Little is known about the prevalence of nutrient deficiencies for infants and toddlers in the United States, making it difficult for healthcare providers to give appropriate dietary guidance to families.
- Nutrient deficiencies in infants and toddlers can have long-term consequences for growth and development as a child and may impact health and wellness as an adult.
- Iodine has not been included in large-scale dietary intake surveys, and little is known about the prevalence or impact of deficiency.
- Intake of nutrients is variable in infants and toddlers and poorly understood, but data suggest children 0–24 months may not be getting enough vitamin D, choline, omega-3 fatty acids, iodine, or iron.

## 6. Supplementation During the First 1000 Days

Both the AAP and the AND recommend all pregnant women consume a multivitamin from conception through lactation [52,168]. It is also recommended that infants and toddlers be supplemented with vitamin D and other nutrients as needed, such as iron. The risk of nutritional inadequacy during pregnancy and the postnatal period leads many American mothers to consume supplements for the health benefits to themselves and their children. Similar concerns for health drive mothers to give supplements to their infants and toddlers. 

### 6.1. Maternal and Fetal Supplementation

#### 6.1.1. B-Vitamins

##### Vitamin B6

Vitamin B6 is generally known for its role in early embryogenesis, however, there is not enough high-quality evidence to support a benefit to vitamin B6 supplementation during pregnancy [70,169]. The evidence suggests there is no significant reduction in risk associated with eclampsia, preeclampsia, or risk of low Apgar scores with supplementation, however, there may be a significant reduction in tooth decay in supplemented mothers [169]. 

##### Vitamin B9 (Folate)

The benefits of folate supplementation on fetal outcomes are well-known. Food fortification with folate resulted in a global, clinically-relevant decrease in congenital anomalies, especially neural tube defects like anencephaly and spina bifida [70]. Additionally, a recent meta-analysis reported iron and folate supplementation have been shown to reduce the risk of LBW and SGA, when compared to iron supplementation alone [170]. Folate supplementation does not impact the risk of preterm birth, stillbirth, miscarriage, maternal mortality, or infant mortality [170,171]. Overall, the evidence in favor of continued fortification and supplementation is strong, however, some attention should be paid to the potential for over-supplementation in this population, since this may impede absorption [172]. 

##### Vitamin B12

There are few relevant clinical trials for B12 supplementation during pregnancy. The few that do exist are designed for geographical regions that have generalized, poor micronutrient status, and may not be applicable to other audiences. These clinical trials conclude that vitamin B12 supplementation improves B12 status in both the mother and the infant [173,174,175], which may be a significant benefit to mothers who are at risk of vitamin B12 deficiency, especially vegetarian and vegan mothers. The trials did not find any conclusive benefit for pregnancy outcomes including neurocognitive development of the infant [175], or preterm birth and risk of LBW and IUGR [173], however, more research is needed, because both trials were conducted starting at 14 weeks gestation, long after neurodevelopment has begun. The benefit for vitamin B12 supplementation, especially as it concerns neurocognitive development, may be found as early as three weeks gestation during embryogenesis and the formation of the neural tube [70]. 

#### 6.1.2. Carotenoids

There is recent evidence to support that maternal supplementation can impact the health of the newborn. Mothers supplemented with carotenoids including 10 mg lutein and 2 mg zeaxanthin from the 28th week of pregnancy gave birth to newborns with lower oxidative stress values, as compared to newborns born to mothers who did not receive such supplementation [176]. Free radical damage caused by oxidative stress can cause structural and functional damage to cells and tissues, leading to many pregnancy complications and abnormalities. Dietary antioxidants such as lutein and zeaxanthin could help protect the mother and infant from these complications.

Lutein and zeaxanthin status of the mother correlates with levels in their newborn infants, so concerted efforts are needed to ensure dietary intakes of women meet the needs of the developing child [177,178]. However, women of childbearing age do not consume adequate amounts of lutein and zeaxanthin through their diet [179,180]. Adding vegetables, such as leafy greens, corn, and bell peppers, to the diet, or prenatal supplements with carotenoids, including lutein and zeaxanthin, can help bridge the dietary gap between the levels required for optimal maternal and infant health, and the intakes being consumed.

#### 6.1.3. Choline

Choline supplementation clinical trials during pregnancy are usually begun between 12 and 18 weeks gestation and run until birth, and sometimes through the postnatal period [181,182,183]. The universal focus of these studies is on some aspect of cognitive development in the neonate including memory [181], processing speed [182], and mental and behavioral health [183]. Choline doses in these studies are usually much higher than the AI (between 750–1000 mg per day) as phosphatidylcholine [181,182,183].

Clinical trials show that choline supplementation during at least the second and third trimester of pregnancy improves processing speed [182] and reduces the risk of sensory development delays [183] in healthy infants. Additionally, choline supplementation shows clinically significant effects in neonates whose mothers have very low choline status [184], suggesting choline supplementation may have a protective effect against developmental delays when maternal choline status is inadequate.

#### 6.1.4. Magnesium

Magnesium supplementation during pregnancy is used to prevent preeclampsia and pre-term birth, but the clinical trials in support of this are limited and conflicting [185]. A recent meta-analysis shows magnesium supplementation reduces the risk of maternal hospital stays during pregnancy, and reduces the risk of low Apgar scores, late-fetal heart decelerations, and meconium-stained amniotic fluid [186]. However, the same meta-analysis saw no significant risk reduction in preeclampsia, SGA, or perinatal mortality [186]. In addition to birth outcomes, a recent clinical trial showed that magnesium supplementation during pregnancy may improve insulin sensitivity and decrease insulin resistance in women with gestational diabetes [187]. 

#### 6.1.5. Omega-3 Fatty Acids

Supplementation of pregnant women with EPA and DHA provides benefits for both the mother and the developing fetus [188,189]. Early studies showed that low consumption of seafood was a strong risk factor for preterm delivery and LBW [190]. This group also showed that maternal plasma concentrations of EPA and DHA during early and mid-pregnancy were strongly associated with early preterm birth, with lower levels of the lipids leading to higher risk [191]. DHA supplementation during pregnancy resulted in longer gestation duration, fewer preterm births, greater mean birth weight, length and head circumference, significantly fewer infants who were admitted to an intensive care unit, and shorter hospital stays for infants born preterm [192,193,194]. Increasing the intake of omega-3 fatty acids is associated with an 11% reduced risk of birth at less than 37 weeks gestation and a 42% reduction in births of less than 34 weeks. 

Some benefits of maternal omega-3 fatty acid supplementation may be found for childhood allergies. In a randomized trial of 706 Australian children, the intervention group receiving fish oil capsules gave birth to infants who were not different in immunoglobulin associated allergic disease compared to the control group [195]. However, there was a decreased percentage of infants with an atopic eczema diagnosis and fewer infants sensitized to eggs in the omega-3 treatment group. A pooled analysis of over 60,000 European and US birth cohorts found no association of fish and seafood consumption with asthma and allergic rhinitis [196].

#### 6.1.6. Trace Minerals

##### Zinc

Zinc supplementation during pregnancy has been extensively studied [70,99,197] and while benefits for the mother have been reported, fetal morbidity and mortality remain largely unaffected [198]. Systematic reviews indicate that a relatively small daily dose of 5–50 mg per day can reduce the risk of preterm birth [198] by as much as 14% [197,199]. This does not translate to an increase in birth size in the infant [199]. Recent evidence also indicates that daily, oral zinc supplementation of 30 mg per day may improve the symptoms associated with gestational diabetes, including increased insulin sensitivity and improved lipid profiles [200]. 

##### Copper

Little is known about copper-containing supplement use in women during pregnancy, however a recent analysis of NHANES data showed that adults who do not regularly consume copper-containing supplements are significantly more likely to have copper intakes that are below the EAR, compared to adults who do consume supplements containing copper [201].

Iron and zinc status have been shown to significantly influence copper status and metabolism, therefore copper is commonly studied in conjunction with these two trace minerals [152,202]. High serum levels of copper are correlated with iron deficiency, and a balance between these two minerals is necessary for proper embryonic growth [152]. High levels of zinc supplementation can cause copper inadequacies in at-risk adults [11], and an observational study in pregnant women showed serum copper status can even be influenced by normal serum zinc levels [202]. 

##### Iodine

The knowledge that iodine deficiency increases the risk for cretinism and other cognitive impairments during early childhood development [32,70,107] has led to the idea that supplementation during pregnancy can reduce the risk of poor cognitive development in infants related to congenital hypothyroidism [203,204]. In areas with severe iodine deficiency, potassium-iodide supplementation in pregnant women reduces the risk of cretinism and improves some aspects of cognitive function [205]. Clinical trials assessing the relationship in areas with mild-to-moderate iodine deficiency (like the United States) or sufficient iodine, do not provide enough high-quality evidence to show that iodine supplementation during pregnancy reduces the risk of poor cognitive development [160,205]. Certain aspects of cognitive functioning in children living in mildly iodine deficient areas may benefit from supplementation [206], but more research is necessary to fully understand this relationship. 

##### Iron

Iron supplementation during pregnancy is recommended by the Centers for Disease Control (CDC) for all women starting at their first prenatal visit [207], however these recommendations are dated. A recent report from the U.S. Preventive Task Force suggests there is little conclusive evidence to recommend routine iron supplementation for healthy, iron-replete women [208]. Clinical trials show iron supplementation reduces the risk of LBW [209], preterm birth, and diagnosis of SGA [210] in children born to anemic pregnant women, and that these results become non-significant in iron-replete women [211]. There is debate about appropriate dosing [209,212] but most clinical trials conclude that iron supplementation is beneficial for women who have IDA or are at risk of IDA [209,210,211,213].

#### 6.1.7. Vitamin A

Vitamin A supplementation during pregnancy can influence both maternal and fetal health, especially in areas with a high risk of deficiency [214]. Supplementation during pregnancy is primarily used to build both maternal and fetal retinol stores as a guard against deficiency both in the prenatal and postnatal periods [215,216]. Supplementation in prenatal studies is usually conducted using large, weekly doses of 7000 RE (retinol equivalents) either as retinyl palmitate or as beta-carotene. In every case, the retinyl palmitate form showed significant benefit over the beta-carotene form [214,217,218]. Beta-carotene showed no additional benefit over placebo [214,217,218]. To maximize benefit to mother and child, supplementation should occur during pregnancy or within the first 48 hours of birth in breastfed newborns [214]. Supplementation in pregnancy has been shown to increase plasma and liver concentrations [217], significantly decrease the incidence of gestational night blindness [217] and to reduce the risk of anemia in pregnant women [219]. 

#### 6.1.8. Vitamin D

Discussion and interest in the benefits of vitamin D during pregnancy have increased dramatically in the last decade and are projected to increase exponentially in the next [220]. To date, there are over 40 randomized clinical trials studying the effects of vitamin D supplementation on maternal or fetal health during pregnancy [113,220,221], however the prevailing conclusion from meta-analyses is that the available data is incomplete [220,222]. Despite this, several themes are emerging for the role of vitamin D supplementation during pregnancy.

The maternal outcomes studied in clinical trials are usually related to the birth process, and not to other facets of maternal health, with the exception of gestational diabetes. One recent clinical trial found that supplementation with 200 IU of vitamin D (as cholecalciferol) improved insulin sensitivity and general glycemia in pregnant women [222,223]. These results were confirmed by two meta-analyses that showed supplementation with 200–600 IU vitamin D (as cholecalciferol) reduces the risk of gestational diabetes in pregnant women [113,220]. However, another meta-analysis showed no difference in risk of gestational diabetes between supplemented and non-supplemented groups [222]. The difference between these meta-analyses may be the timing of supplementation. Von Websky, et al., argued the data showing the strongest benefit of supplementation on gestational diabetes risk are from studies that begin supplementation pre-pregnancy or within the first 6 weeks of pregnancy [117].

Vitamin D supplementation during pregnancy has also been shown to reduce the risk of preeclampsia in high-risk women [117,222], although the data is limited, and may be specific to at-risk populations. There is insufficient data to draw conclusions about the role of vitamin D supplementation for risk of caesarean birth, preterm birth or stillbirth [113,117,220,222].

In addition to maternal health, vitamin D supplementation during pregnancy impacts fetal health. Meta-analyses of randomized controlled trials report that daily maternal vitamin D supplementation between 200–600 IU reduces the risk of LBW [220,222] and may also impact skeletal growth by increasing infant length and head circumference [222]. There is insufficient data to draw conclusions about the role of vitamin D supplementation for the risk of other fetal morbidities or neonatal death [222]. 

**Key Takeaways:**

- Supplementation of fat-soluble vitamins, A and D, during pregnancy may influence maternal and fetal health, especially in areas with a high risk of deficiency.
- Among the trace minerals, zinc and iron supplementation during pregnancy have been extensively studied. Oral zinc supplementation at a dose of 5–50 mg/day shows some benefit for the mother, however more research is needed to understand its effects in baby.
- Iron supplementation can reduce the risk of poor birth outcomes in anemic women.
- Among the B vitamins, the most is known about the benefits of folate (vitamin B9). A recent meta-analysis showed folate supplementation can reduce the risk of LBW and SGA
- Supplementation with omega-3 fatty acids during pregnancy has several benefits for the mother and developing child. These include decreased risk for preterm birth, and a decrease in childhood allergies.
- Choline supplementation during pregnancy may help to optimize cognitive development in the fetus.

### 6.2. Maternal Postnatal Supplementation

#### 6.2.1. Vitamin A

Postnatal supplementation of vitamin A for maternal benefit is not well-documented, and relevant data are mostly given as additional points of interest in child-based studies. Weekly, oral supplementation of 7000 RE of retinyl palmitate during pregnancy significantly reduced the prevalence and incidence of bacterial vaginosis at three months postpartum compared to supplementation with equivalent beta-carotene or placebo [215]. The results of the data from this study show that vitamin A supplementation during pregnancy may have some immune effect in the postpartum period, although neither the relationship nor the mechanism of action is known at this time [215]. 

Additional evidence for benefits of vitamin A supplementation show that postpartum serum, liver, and breast milk retinol levels increase when vitamin A is supplemented either during pregnancy or immediately in the postpartum period (up to 48 h after birth) [215,218,224]. Postpartum supplementation in these studies show a single, large dose of 200,000 IU retinyl palmitate increases retinol availability in plasma and breast milk, but that no additional benefit is seen from 400,000 IU [218,224,225,226].

#### 6.2.2. Vitamin D

The role of vitamin D supplementation in the postpartum period is just beginning to be understood [227,228,229]. Of the multiple areas of interest for vitamin D supplementation, including postpartum depression [228], clinical data only exists to support bone health. One promising clinical trial showed that vitamin D in combination with calcium supplementation was able to decrease bone loss due to breastfeeding during the postpartum period [229]. 

### 6.3. Supplementation During Breastfeeding

Certain nutrients are difficult to acquire through diet alone, so many women turn to supplements to ensure adequate nutrient status in breast milk [230]. Nutrients that may require supplementation include iron, iodine, folic acid, and vitamin D [230,231]. Information is not always available for micronutrient supplementation during lactation, therefore only nutrients with substantial evidence are discussed.

#### 6.3.1. Calcium

Calcium content in breastmilk is dependent on maternal calcium and vitamin D status [232]. Low serum calcium or vitamin D results in lowered calcium content [232,233]. Breastfeeding mothers typically deposit 200 mg of calcium into breastmilk per day, and it is thought this calcium is derived from increased bone resorption [74]. The calcium loss in bones typically resolves after weaning [74], and there is no conclusive evidence as to whether this leads to long-term bone mineral density complications [234,235]. The role of calcium supplementation during lactation is not well documented.

#### 6.3.2. Carotenoids

The macular and brain carotenoid levels in infants following delivery are a result of infant intake from the colostrum and mother’s milk, or infant formula; the characteristic yellow color of colostrum is due to the carotenoids present [236,237,238]. A recent study found that the colostrum of mothers that gave birth to premature infants contained lower concentrations of carotenoids as compared to mothers of full-term infants; however, lutein levels were not different, which authors speculate could be related to the significant role of lutein in development of the infant retina and brain [239]. Several studies have analyzed the carotenoid content in human breast milk across diverse populations worldwide [237,240,241,242,243,244,245,246,247,248,249,250]. These findings demonstrate a strong correlation between dietary intake of carotenoids and the content found in breast milk [178,251,252].

The impact of carotenoid supplementation on breastfeeding women and their infants was assessed in a randomized, double-blind, placebo-controlled trial [253]. The study revealed that supplementation with lutein beginning at 4 to 6 weeks postpartum at both a 6 mg and 12 mg per day dose for 6 weeks not only significantly increased the lutein content in the mother’s serum and breast milk, but also significantly increased the lutein and zeaxanthin concentration in infant serum. The direct correlation between concentrations of breast milk lutein and zeaxanthin with their daily intake, along with the evidence that lutein and zeaxanthin supplementation for lactating mothers can impact quantities in their infants, emphasizes the importance of these key nutrients, not only throughout the duration of pregnancy, but also during lactation, to ensure ample quantities for visual and cognitive development of the infant.

#### 6.3.3. Choline

Choline requirements continue to be high in postnatal women because of the increased choline needs in breast milk [142]. Free choline is converted mostly to phosphocholine and glycerophosphocholine in the mammary glands in large quantities [82], and maternal choline status from food intake has a moderate effect on choline and phospholipid content in breast milk [142]. It is unknown if choline supplementation can improve the choline status in breast milk, or if indirect supplementation by the mother can improve the choline status in the infant through breast milk. 

#### 6.3.4. Iodine 

Maternal iodine status can have a significant impact on infant iodine status, if the infant is breastfed, and especially if exclusively breastfed [104]. Newborns who are born deficient in iodine may have difficulties absorbing an iodine supplement because of their immature digestive systems, and evidence suggests a maternal iodine supplement can transfer more iodine than direct supplementation to the infant [254]. Supplementation trials studying the benefits of iodine supplementation during breastfeeding do not exist for either mother or infant, however, an observational study from the United States shows 47% of breastfeeding women demonstrate insufficient iodine status in their breast milk [157].

#### 6.3.5. Omega-3 Fatty Acids

There is a direct and linear correlation between maternal DHA intake and breast milk DHA content [255]. Although the worldwide level of breast milk DHA has been estimated to be 0.32% [256], daily supplementation of 1.3 g DHA can increase breast milk DHA levels [255]. Increasing breast milk DHA leads to a dose-dependent increase in infant plasma and erythrocyte phospholipid DHA [257]. 

#### 6.3.6. Vitamin A

Supplementation trials for vitamin A are typically conducted in areas where vitamin A deficiency is widespread [216,218] and focus on achieving additional vitamin A content in breast milk with mega-doses of vitamin A [218]. These studies show that in at-risk or deficient women large doses up to 200,000 IU vitamin A as retinyl palmitate can improve vitamin A content in breast milk, and subsequently in infants without adverse effects. A meta-analysis of vitamin A supplementation studies in lactating women found no additional benefit to maternal or infant health beyond improving serum and breast milk levels [218]. The results of these studies are not necessarily applicable to women residing in places like the United States where there is vitamin A sufficiency. The benefit of vitamin A supplementation in lactating women who are vitamin A sufficient is unknown. 

#### 6.3.7. Vitamin B12

Early infant B12 status is heavily influenced by maternal B12 status [126] both during pregnancy and while breastfeeding [126]. Maternal supplementation of vitamin B12 while breastfeeding has shown to improve B12 levels in colostrum and breast milk [173,174] in vegan and non-vegan mothers and may help reduce the risk of deficiency in infants during the first months of life.

#### 6.3.8. Vitamin D

Breast milk contains very little of the active form of vitamin D (1,25(OH)_2_D_3_); consequently, infants must synthesize their own from sunlight and other forms of vitamin D, including 25(OH)D [258]. Infants have some vitamin D stores at birth, but, by 4 to 6 months of age, these supplies are depleted, and vitamin D supplementation is recommended [113]. Supplementation can either be given to the infant as liquid drops [113] or to the mother, if the infant is breastfed [233]. Maternal supplementation with the 25(OH)D increases serum and breast milk 25(OH)D, but does not impact the calcium content of breast milk [233]. Supplementation trials show that large doses of 6000 IU or higher vitamin D as 25(OH)D are necessary to ensure breastfed infants receive the appropriate amounts of vitamin D precursor through breast milk [114,259]. These levels are significantly higher than the Institute of Medicine (IOM) recommendations of 600 IU per day [12] for lactating women, so careful consideration on a case-by-case basis and further study is warranted when making recommendations for lactating women. 

**Key Takeaways:**

- Very little information has been reported in the literature on maternal postnatal health or the potential benefits of supplementation. There is very little information for healthcare providers to help guide mothers in their post-birth recovery or their nutritional transition to motherhood.
- Breast milk lacks key nutrients, including iron, iodine, folic acid and vitamin D, and may have low levels of omega-3 fatty acids. Women who are exclusively breastfeeding their infants should consider taking a supplement to achieve replete status.

### 6.4. Infants and Toddlers Supplementation, 0–24 Months

#### 6.4.1. Carotenoids

Multiple studies have supplemented infants with lutein and zeaxanthin, demonstrating these nutrients are safe and effective in increasing plasma concentrations of lutein and zeaxanthin and reducing oxidative stress and inflammation [260,261,262,263,264,265,266]. Plasma lutein levels in infants have been shown to correlate with the saturated response amplitude in rod photoreceptors, suggesting that lutein helps protect photoreceptors and potentially impacts retinal development through its ability to reduce inflammation [266]. Lutein and zeaxanthin also act as a visual filter. Their ability to absorb blue wavelengths of visible light not only protect against the damaging effects of sunlight but also light emitted from electronic devices. A study of over 300 children ages 6 months to 4 years found most children were using mobile devices daily by age two [267]. Carotenoid supplementation in college-aged adults with high screen time exposure (at least 6 h of viewing time daily) improved visual function and reduced symptoms associated with excessive screen time [268]. While this study was conducted in adult subjects, it demonstrates the ability of lutein and zeaxanthin to improve the visual and physical issues related to the dramatic increase in digital exposure at all ages. 

Studies in animal models have revealed that carotenoids are very important for proper retinal development [269,270,271]; however, lutein’s role in neural development is a relatively new area of research. There is evidence suggesting a preferential uptake of lutein into infant neural tissue. NHANES III data for infants 2–11 months of age indicate lutein and zeaxanthin account for roughly 12% of total carotenoids consumed in the diet, and beta-carotene and lycopene together account for approximately 70% [11]. However, levels in the infant brain are quite different in that lutein and zeaxanthin represent 74% of all carotenoids present, whereas beta-carotene and lycopene account for only 20% [272]. Interestingly, the relative contribution of lutein to the total carotenoids in the brain of infants is twice that found in the brains of adults [273]. This suggests that lutein may play a role in regulating brain volume or structural growth in the development or remodeling of neurons [274]. Additional evidence supporting lutein’s role in cognitive development comes from the finding that higher lutein levels in maternal breast milk at 3–5 months postpartum were related to better infant recognition memory [275].

#### 6.4.2. Omega-3 Fatty Acids

Supplementation of the infant diet with omega-3 fatty acids can lead to better infant cognitive and visual development, due to the importance of DHA in nervous system function. Infants can be supplemented through either direct addition to their formula or maternal supplemented breast milk. Visual acuity has been a frequently used outcome for these supplement studies and can be measured behaviorally or electrophysiologically. Healthy preterm infants given fish oil supplements in their formula had improved visual acuity when measured at 2 months of age, but not at longer time points [276]. It was further found that full term infants supplemented with DHA and AA had better sweep visual-evoked potential acuity at several time points and extending up to one year of age; these results correlated with plasma DHA levels [277,278,279]. In a subsequent study, these authors found better visual acuity and stereo acuity in supplemented infants that correlated with their plasma DHA content [278]. Smithers, et al., subsequently found better sweep visual-evoked potential acuity in high-risk infants, and similar results were reported in DHA-supplemented, preterm infants [280,281].

A 7-point increase in IQ was reported with the Bayley’s Mental Development Index (MDI) in full-term infants, whose formula included DHA and AA [279]. However, no significant effect of DHA supplementation on Bayley’s MDI was observed in 18-month-old preterm infants overall [282]. Secondary analyses indicated that girls displayed a higher MDI score in the high DHA group relative to the lower DHA supplemented group; moreover, there was an observed benefit of the higher DHA supplementation in infants weighing less than 1250 g at birth. 

Longer term cognitive outcomes for infants fed AA and differing levels of DHA in their infant formulas have also been reported [283]. However, while there were significant positive effects on rule learning and inhibition tasks, Peabody Picture Vocabulary, and Weschler Primary Preschool scales of Intelligence were observed for the supplemented groups in children from 3–5 years, no benefit for standardized tests of language and performance at 18 months of age were reported [283]. 

A decline in bronchopulmonary dysplasia in boys and a reduction in reported hay fever in all infants at 18 months was observed when high-dose DHA was fed to preterm infants. No effects were found for asthma, eczema or food allergy [284]. A reduction in both the occurrence and odds ratio of allergic diseases, upper respiratory infections, wheezing, and asthma in the first year of life, as well as a delay in the time to the first allergic illness and skin allergic illness was reported when healthy, full-term infants were fed AA and DHA supplemented formula [285,286]. A dose-dependent relationship between DHA toddler supplementation and incidence of respiratory illness over 60 days has also been established [60]. 

#### 6.4.3. Trace Minerals

##### Zinc

In zinc-deficient children under five years old there is some evidence that mild supplementation of 10–20 mg elemental zinc can reduce the risk of diarrhea incidence, stool frequency and diarrhea duration [287]. There is also evidence that zinc supplementation can reduce the risk of respiratory infection [287,288] and pneumonia [99,288] in the same population. Recent meta-analyses of both observational data and clinical trials have shown that zinc supplementation can increase weight, height, and weight-for-age z-score in term infants [288,289,290], and that supplementation has the largest impact when begun before two years of age [289]. However, the authors conclude that multiple micronutrient supplementation is still superior to zinc supplementation alone in this age group [290].

##### Iron

Iron supplementation in infants and young children 0–2 years is usually in the form of food fortification in formula, prepared baby foods, and fortified grains and cereals [291,292]. In children 0–2 years old, taking supplements, 16.2% were taking supplements containing iron [91]. Clinical trials that assess traditional supplementation as a single-nutrient oral dose are limited and are primarily conducted in low-income countries with high levels of IDA. These supplementation studies found that daily, oral iron supplementation between 6 and 12 months reduced the risk of IDA in infancy [292,293,294,295,296,297] and improved psychomotor development scores in anemic babies at 12 months old [293]. However, there was no significant neurocognitive improvement reported in iron-replete babies at 9 months [294], 12 months [293], 18 months [295], 6 years [296], or 9 years of age [297]. The research indicates the most significant benefits of iron supplementation are seen in high-risk groups [70,292] with infants who have, or are at high-risk for, IDA [293,296], or who live in areas where there is a high prevalence of iron deficiency [70]. All current research agrees the relationship between iron, general development, and neurocognitive development is not fully understood, and further research is needed [70,292,293,294,295,296,297]. 

#### 6.4.4. Vitamin A

Supplementation with vitamin A may have the most benefit to the child when begun in utero [217,226,298]. No childhood outcomes were improved when the supplementation was begun in the postpartum period [215,225]. In trials completed outside the USA, single, large doses of 50,000 IU vitamin A, given to the infant immediately after birth, failed to improve the mortality rates of the children at one year of age [299]. Similarly, large doses of vitamin A given to the mother immediately postpartum failed to improve childhood mortality [299]. It is unclear whether vitamin A supplementation, either as large, single doses or as smaller, repeat doses, throughout the first two years of life can improve health outcomes for children. 

#### 6.4.5. Vitamin D

It is generally well accepted that vitamin D supplementation promotes optimal bone growth and prevents rickets in infants [300], and newer clinical trials continue to find this result [301,302]. However, there is some debate about the optimal supplemental dose for infants. The current recommendation of 400 IU [300] is found to not be sufficiently high to achieve vitamin D status in serum [302,303,304] but longitudinal studies find no additional benefit of dosages higher than 400 IU on bone mineral status and other developmental outcomes between 3 and 6 years of age [301,302]. Observational studies find that vitamin D intake in infants is inadequate [305] and, despite strong recommendations from governing bodies [300], adherence to supplementation requirements is less than 20% in infants less than 6 months old [306]. 

**Key Takeaways:**

- Clinical trials of nutrient supplementation show that recommended intakes for vitamins and minerals in infants and toddlers can be benefited from further review, including vitamin D, omega-3 fatty acids, iron, and carotenoids.
- The benefits of supplementation for copper, iodine, vitamin B6, folate, vitamin B12, magnesium and choline are poorly understood in infants and toddlers, 0–2 years old.
- Carotenoids are currently used in infant formula, with no adverse health reports.
- For deficient children, nutrient supplementation can impact long-term growth and development, and may influence health and wellness into adulthood.

## 7. The Role of the Microbiome in Pregnancy, the Postnatal Period, and the Growing Child

The first 1000 days of life are a critical period of development, and all aspects of environment may play a role in shaping future health, including the microbiome [307]. Bacterial colonization may begin early, during fetal development. It has been previously thought that the uterus is sterile and the first encounter of the infant with microbes happens during delivery. However, there is recent literature that has identified microbes from placenta [308], amniotic fluid [308], umbilical cord [309], and meconium [310]. Microbiome changes in composition and diversity begin in the first trimester and continue to change through the third trimester [311]. These changes are usually associated with metabolic syndrome and disorders, however, in the case of pregnancy they are considered to be beneficial for pregnant women, since they would support the growth of the fetus and also potentially help with energy demands during the lactation period [311].

Since pregnancy itself is associated with transformation of the gut microbiota towards what is usually considered an obesogenic microbiota, there are also studies in which the impact of gestational diabetes (GDM) and/or obesity on gut microbiota has been assessed. GDM has been shown to be associated with further changes in the gut microbiota; Actinobacteria have been shown to be enriched, similarly to species within genera *Collinsella*, *Rothia,* and *Desulfovibrio*. Moreover, even after adjustment for BMI before pregnancy, five OTUs were different between women with GDM and healthy pregnant women of which *Butyricicoccus* was negatively associated with insulin sensitivity and *Akkermansia* spp. was associated with lower insulin sensitivity [312]. 

After delivery, it takes several months for the mother’s gut microbiota to return to the pre-pregnancy state. It has been shown that delivery and lactation don’t significantly alter the gut microbiota, at least for the first month [313]. Moreover, the gut microbiota of mothers with GDM did not return to a “normal” state, even after 8 months postpartum [312]. One important microbial population after the delivery is milk microbiota. It has been proposed that a bacterial entero-mammary pathway enables the milk microbiota to exist. Streptococcaceae, Staphylococcaceae, and Bifidobacteriaceae families have been reported to form the core milk microbiota [314]. However, the milk microbiota varies between different geographical locations and delivery mode. For example, Chinese women have been shown to have a high abundance of Actinobacteria, whereas Spanish women have been shown to have a high abundance of Bacteroidetes. Moreover, women having a cesarean birth have been shown to have a high abundance of Proteobacteria [315]. 

Vaginal, oral, and skin microbiota of the mother will also have an impact on the seeding of the infant’s microbiota. Healthy vaginal microbiota is dominated by lactobacilli, and, unlike other microbial populations within the human being, lower α-diversity is associated with healthier vaginal microbiota. Moreover, studies have shown that disturbed vaginal microbiota may impact the length-for-age *Z*-score (LAZ), and therefore influence the growth of the infant [316]. The importance of oral and skin microbiota in pregnancy has been studied less, but they have been shown to affect the initial colonization of bacteria within an infant gut [317]. 

**Figure 3:**
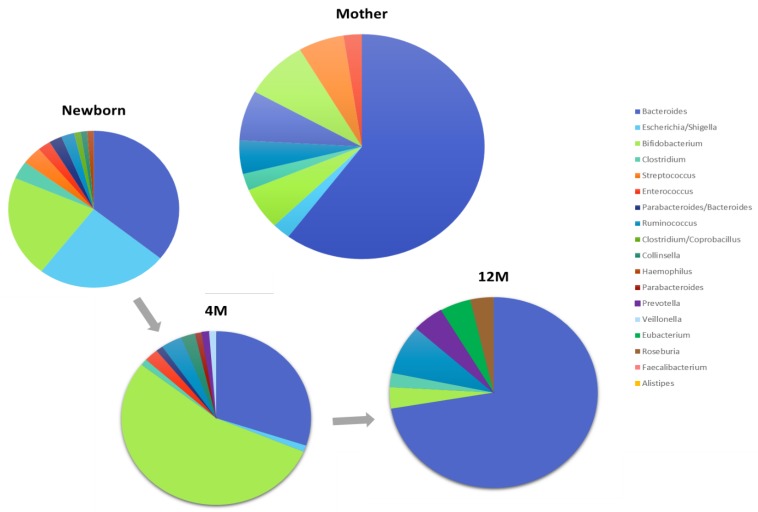
The most abundant bacterial genera of mothers and their infants at different ages (newborn, 4 months, and 12 months). The values are presented as percentage of number of samples with this genus as the most abundant genus. The figures were adapted from Bäckhed et al., 2015 [319].

Bacterial colonization of the infant gastrointestinal tract is influenced by mode of delivery, prematurity, type of feeding (breast feeding vs. formula feeding), antibiotic treatment of the child or the mother, lifestyle, and geographical location [318]. The earliest colonizers are usually facultative anaerobic bacteria such as Enterobacteriaceae, Streptococcaceae and Staphylococcaceae, whereas later colonizers tend to be strict anaerobes, e.g., *Bifidobacterium* spp., *Bacteroides* spp., and clostridia, regardless of the infant’s geographical origin and methods used for the detection [311,317,319,320] (Figure 3). It has also been shown that the mother-to-infant microbial transmission has been compromised in infants born by caesarean, since only 41% of the fecal infant early colonizers (at species level) were found from the mother’s fecal microbiota, whereas, in the case of vaginal delivery, 72% of the species were found from the mother’s fecal microbiota [319]. Caesarean birth has also been shown to alter microbial β-diversity, as compared to vaginally-delivered infants. Moreover, in a recent study it was shown that a decrease in Bacteroidetes in caesarean infants was also associated with an altered metagenomic landscape during the first year of life. Since *Bacteroides* spp. are an important species with regards to regulation of intestinal immunity, these changes could have long-lasting health implications [320]. 

Immediately after birth, within the first 24 h, high microbial species diversity in the infant have been observed, but this decreases during the first week of life [317]. The fecal microbiota of vaginally delivered babies has been shown to be enriched in *Bacteroides* spp., *Bifidobacterium* spp., *Parabacteroides* spp., and *Escherichia* / *Shigella* spp. (Figure 3), whereas the fecal microbiota of babies delivered by Caesarean birth has been shown to be enriched with *Enterobacter* spp., *Haemophilus* spp., *Staphylococcus* spp., and *Veillonella* spp. 

Importantly, some of the early colonizers are transient, for example *Haemophilus parainfluenzae* and *Prevotella melanonigenica*, were found in the infant fecal sample at day one but were not found at subsequent sampling points [319]. Since these bacterial species are not usually associated with fecal/gut microbiota, they most likely originated from body sites of the mother than the gastrointestinal (GI) tract [317]. Bacterial species that are associated with the GI tract, e.g., *Bacteroides vulgatus*, *Bifidobacterium longum,* and *Bifidobacterium breve*, were found throughout the follow-up period of 4 months [319]. In addition, vaginal species, which totaled up to 16% of infant fecal microbiota at day 1, were under detection limits at 1 week of age [317]. 

By the end of the first year of life, when the child has already started to eat the same foods as the adults and ceased breastfeeding, the gut microbiota starts to converge towards a profile characteristic of the adult microbiota (Figure 3). However, the fecal bacterial diversity is still lower [319]. In a cohort from the U.S., it has been shown that the relative abundance of *Bifidobacterium* spp., *Ruminococcus* spp., *Veillonella* spp., and Erysipelotrichaceae decreases, whereas the relative abundance of *Faecalibacterium* spp. and Clostridiales increases between 1 year of age and 2 years of age [320]. By the end of the second to third year, the phylogenetic composition evolves even more towards the adultlike composition [320]. Moreover, the gut microbiota will continue to evolve. It has been shown in children and adolescents that, even though the microbiota starts to resemble that of adults, there are still differences from the adult microbiome in the microbial diversity and microbial pathways throughout childhood [321].

**Key Takeaways:**

- The gut microbiota changes during pregnancy and continues to evolve during the postpartum phase. Changes in microbial diversity and composition have been noted from the first to third trimesters.
- The mother’s fecal, vaginal, oral and skin microbiota have a direct impact on the infant’s microbiota
- Bacterial colonization of the infant’s gastrointestinal tract is influenced by mode of delivery (vaginal vs. caesarean)
- By the end of the first year of life, gut microbiota begins to converge towards an “adult like” profile. Microbiome changes continue through childhood.

## 8. Supplement Safety

The overall safety of dietary supplements produced with good manufacturing practices and stored and consumed within the recommended time period is not in question. Furthermore, they have a long history of safe use in foods and OTC medications. The IOM, European Commission’s Scientific Committee on Food (EC SCF) and its successor EFSA, the UK’s Expert Group on Vitamins and Minerals (EVM), industry groups, and peer-reviewed publications have all reviewed and published risk assessments for one or several of the vitamins and minerals (including trace elements) used in most reputable dietary supplement products. These agencies have all concluded that vitamin and mineral intake is safe but should not exceed the UL. Supplemental intakes of several nutrients provide clearly established benefits for many people, most obviously for those in specific age and gender groups, e.g., children and pregnant women.

## 9. Conclusions

The first 1000 days of life represents a critical period for healthy growth and development. This review has found that, while all essential nutrients are required to support a healthy pregnancy, eight key nutrients, including carotenoids (lutein + zeaxanthin), choline, folate, iodine, iron, omega-3 fatty acids and vitamin D, are necessary throughout the stages of gestation, during the postpartum period, and through the 2nd birthday. This review has also identified that little information exists regarding the prevalence of nutrient deficiencies for infants and toddlers in the USA. Nutrient deficiency in this age group is of concern because it can have long-term consequences on growth and development and may also impact wellness as an adult. Therefore, it is critical that we begin to close this knowledge gap by studying children more. 

In terms of supplementation, very little information has been reported in the literature on maternal postnatal health. This makes it challenging for healthcare providers to guide post-partum mothers on their nutritional transition to motherhood. Similarly, for infants and toddlers, research on nutrient supplementation is limited, and more work is especially needed on nutrients that play key roles in visual and cognitive development, such as the carotenoids, iron, vitamin D, and omega-3 fatty acids. 

Finally, an evolving area of research relative to this review topic, is the role of the microbiome from pregnancy through the child’s second birthday. From this review, we’ve learned that the gut microbiota adapts during pregnancy and continues to evolve during the postpartum phase. The bacterial colonization of the infant’s gastrointestinal tract is influenced by the mode of delivery, however, by the end of the first year of life, the gut microbiota appears more adult-like.

While much remains to be discovered in the areas of nutrient supplementation, nutrient deficiencies, and the changing gut microbiota, expectant mothers should continue to work with nutrition gatekeepers and qualified healthcare practitioners. Achieving a diet plan that provides flexibility in planning while still meeting nutrient requirements for carotenoids (lutein + zeaxanthin), choline, folate, iodine, iron, omega-3 fatty acids and vitamin D is critical. Expectant mothers who fall short on these nutrients may consider taking a supplement to help fill their dietary gap.

## Figures and Tables

**Table 1:** Overview of Major Physiological Changes that Occur During a Healthy Pregnancy.

Organ System	Description of Changes with Pregnancy
Cardiovascular	Cardiac output increases 20% by 8 weeks gestation and maternal heart rate increases by 10–20 bpm Blood pressure decreases in the first and second trimesters, but increases during the third. Cardiac output increases during labor (15% in stage 1 labor, 50% in stage 2 labor, with uterine contractions leading to an auto-transfusion of blood (300–500 mL) back into circulation)
Urinary	Renal vasodilation occurs and renal plasma flow and glomerular filtration rate (GFR) increases compared to non-pregnant levels, by 40%–65% and 50%–85%, respectively As GFR increases, serum creatinine and urea concentrations decrease to 44.2 μmol/L and 3.2 mmol/L Increases in renal blood flow cause an overall increase in renal size by 1–1.5 cm, reaching the maximal size by mid-pregnancy Kidney, pelvis, and calyceal systems dilate due to mechanical compressive forces on the ureters Alterations in tubular handling of wastes and nutrients occur Less effective reabsorption of glucose and variability in glucose excretion
Endocrine	Levels of human chorionic gonadotropin (hCG), estrogen, and progesterone peak during pregnancy and may cause increased nausea and vomiting Electrolyte imbalances, dehydration, weight loss, or vitamin and mineral deficiencies may occur in severe cases of nausea and excessive vomiting (hyperemesis gravidum)
Gastrointestinal	Mechanical changes in the alimentary tract occur due to the growing uterus; the stomach is displaced upwards leading to increased intra-gastric pressure Other gastrointestinal problems may also occur during pregnancy, including heartburn, constipation and incontinence
Hematological	Plasma volume increases by 50% by week 34 of gestation and is proportional to the birthweight of the baby Hemoglobin, hematocrit, and red blood cell count decrease Requirements for iron, folate, and vitamin B12 increase to accommodate hemoglobin synthesis by the mother and the growing fetus Production of clotting factors (VIII, IX and X) increases to prepare for hemostasis following delivery
Respiratory	Demand for oxygen increases due to a 15% increase in metabolic rate and a 20% increased consumption of oxygen Feelings of breathlessness without hypoxia are common in the third trimester, but may occur anytime during pregnancy
Skeletal	Maternal bone turnover is low in the first trimester, but increases during the third trimester when fetal calcium needs are highest

Information for this table collected from [3,4].

**Table 2:** Comparison of Dietary Recommended Nutrient Intakes (DRIs) for Pregnant, Lactating and Non-Pregnant Women, Age ≥19 years.

Nutrient	DRI (Pregnancy) DRI (Lactation)	DRI (Non-Pregnancy)	Examples of Common Dietary Sources (Listed Alphabetically)
Carbohydrate	175 g/day 210 g/day	130 g/day	Fruits, legumes, low-fat dairy products, vegetables (starch and non-starchy), whole grains
Total Fiber	28 g/day * 29 g/day *	25 g/day *	Fruits, legumes, vegetables, whole grains
Protein	71 g/day 71 g/day	46 g/day	Animal sources: Beef, chicken, dairy products, eggs, pork, seafood, turkey Plant sources: Legumes, nuts, quinoa, seeds, soy
Total Fat	No DRI, 20%–35% of total calories suggested		Limit saturated and *trans*-fat, increase consumption of polyunsaturated fatty acids
Linoleic Acid (Omega-6)	13 g/day * 13 g/day *	12 g/day *	Nuts, seeds, vegetable oils (including soybean, safflower and corn oil)
alpha-Linolenic Acid (Omega-3)	1.4 g/day * 1.3 g/day *	1.1 g/day *	Fatty fish, oils (including flax seed). Smaller amounts found in poultry, meats and eggs
Vitamin A	770 μg RAE/day 1300 μg RAE/day	700 μg RAE/day	Apricots, broccoli, carrots, fortified milk and eggs, kale, mangoes, margarine, sweet potatoes
Vitamin C	85 mg/day 120 mg/day	75 mg/day	Citrus fruits, kiwifruit, strawberries, vegetables (red pepper, green pepper, broccoli, Brussels sprouts, cabbage)
Vitamin D	600 IU/day 600 IU/day	600 IU/day	Eggs, fatty fish, fortified foods such as orange juice and milk
Vitamin E	15 mg/day 19 mg/day	15 mg/day	Nuts, plant-based oils, seeds
Vitamin K	90 μg/day * 90 μg/day *	90 μg/day *	Broccoli, green beans, kale, peas, spinach, vegetable oils (canola, soy)
Vitamin B6	1.9 mg/day 2.0 mg/day	1.3 mg/day	Fish, meat, poultry and whole grains including oats
Vitamin B12	2.6 μg/day 2.8 μg/day	2.4 μg/day	Dairy products, eggs, meat, poultry, seafood
Choline	450 mg/day * 550 mg/day *	425 mg/day *	Beef and chicken, eggs (with yolk), mushrooms, salmon, wheat germ
Folate	600 μg/day 500 μg/day	400 μg/day	Beans, dark green vegetables (including spinach and asparagus), fortified cereals, fortified juices (including orange juice), nuts
Calcium	1000 mg/day 1000 mg/day	1000 mg/day	Cheese, green leafy vegetables (including broccoli, kale, and cabbage), milk, yogurt
Iodine	220 μg/day 290 μg/day	150 μg/day	Dairy products, fish, iodized salt, seaweed
Iron	27 mg/day 9 mg/day	18 mg/day (19–50 years.) 8 mg/day (51 years+)	Heme sources: Fish, meat, poultry, seafood Non-heme sources: Fortified cereals, nuts, seeds, spinach
Zinc	11 mg/day 12 mg/day	8 mg/day	Nuts, legumes, meat, seeds, shellfish
Lutein	--† --†	--†	Cooked leafy greens (including spinach and kale), egg yolks
Zeaxanthin	--† --†	--†	Corn, yellow and orange peppers

Abbreviations: DRI = Dietary Reference Intake, g/day = grams per day, IU = International Units, kg = kilograms, mg/day = milligrams per day, PUFA = polyunsaturated fat, RAE = retinol activity equivalents, μg/day = micrograms per day, yrs. = years. * Represents an Adequate Intake (AI). † There are no daily recommended values established in the United States. Information for this table collected from [7,8,9,10,11,12].

**Table 3:** Recommended Daily Nutrient Intakes for Children, Age 0–24 months.

Nutrient	Infants 0–6 Months	Infants 7–12 Months	Children 1–3 Years
Carbohydrate	60 g/day *	95 g/day *	130 g/day
Total Fiber	ND	ND	19 g/day *
Protein	9.1 g/day *	11.0 g/day	13 g/day
Total Fat	31 g/day *	30 g/day *	ND
Linoleic Acid (Omega-6)	4.4 g/day *	4.6 g/day *	7.0 g/day *
Alpha-Linolenic Acid (Omega-3)	0.5 g/day *	0.5 g/day *	0.7 g/day *
Vitamin A	400 μg RAE/day *	500 μg RAE/day *	300 μg RAE/day
Vitamin C	40 mg/day *	50 mg/day *	15 mg/day
Vitamin D	400 IU/day *	400 IU/day *	600 IU/day
Vitamin E	4 mg/day *	5 mg/day *	6 mg/day
Vitamin K	2.0 μg/day *	2.5 μg/day *	30 μg/day *
Vitamin B6	0.1 mg/day *	0.3 mg/day *	0.5 mg/day
Vitamin B12	0.4 μg/day *	0.5 μg/day *	0.9 μg/day
Choline	125 mg/day *	150 mg/day *	200 mg/day *
Folate	65 μg/day *	80 μg/day *	150 μg/day
Calcium	200 mg/day *	260 mg/day *	700 mg/day
Iodine	110 μg/day *	130 μg/day *	90 μg/day
Iron	0.27 mg/day *	11 mg/day	7 mg/day
Zinc	2 mg/day *	3 mg/day	3 mg/day
Lutein	--†	--†	--†
Zeaxanthin	--†	--†	--†

Abbreviations: DRI = Dietary Reference Intake, g/day = grams per day, IU = International Units, kg = kilograms, mg/day = milligrams per day, ND = not determined, PUFA = polyunsaturated fatty acids, RAE = retinol activity equivalents, μg/day = micrograms per day, yrs.=years. * Represents Adequate Intake (AI). † Daily recommended values not established in the United States. Information for this table collected from [7,8,9,10,11,12].

## Supplementary Materials

The following are available online at https://www.mdpi.com/2072-6643/11/12/2891/s1, Figure S1: Example Search Terms for Choline.
