# Supplementary material for: Nutritional Gaps and Supplementation in the First 1000 Days
Source: Nutrients. 2019 Nov 27;11(12):2891. doi: 10.3390/nu11122891 (PMC6949907; doi:10.3390/nu11122891)
Supplement: Supplementary file 1 [file nutrients-11-02891-s001.pdf]

Supplemental Figure 1. Example Search Terms for Choline

- (Prenatal OR pregnant OR pregnancy) AND maternal care + choline
- Physiol change pregnancy + choline
- (Physiology OR Physiologic OR Physiological) AND postnatal changes + choline
- (Physiology OR Physiologic OR Physiological) AND (breast OR breastfed OR breastfeed OR breastfeeding) + choline
- (Physiology OR Physiologic OR Physiological) AND (fetal OR fetus OR gestation) + choline
- (Physiology OR Physiologic OR Physiological) AND (neonate OR newborn OR baby OR infant OR toddler) + choline
- (fetal OR fetus OR gestation OR neonate OR newborn OR baby OR Infant OR Toddler) AND (development OR milestones) + choline
- (pregnancy OR pregnant OR prenatal OR perinatal) AND (nutrition OR nutritional) (requirements OR optimal) + choline
- (breast OR breastfed OR breastfeed OR breastfeeding OR lactat) AND (nutrition OR nutritional) (requirements OR optimal) + choline
- (fetal OR fetus OR gestation) AND (nutrition OR nutritional) requirements + choline
- (neonate OR newborn OR infant) AND (nutrition OR nutritional) requirements -preterm + choline
- Nutr change pregnancy + choline
- (infant OR toddler) AND nutrition optimal + choline
- first 1000 days nutrition + choline
- first one thousand days nutrition + choline
- (Pregnancy OR pregnant OR maternal OR mother) AND (gap OR deficiency) -knowledge -education + choline
- (Postnatal OR postpartum) AND nutrition AND (gap OR deficiency) -knowledge -education + choline
- (breast OR breastfed OR breastfeed OR breastfeeding) AND nutrition AND (gap OR deficiency) -knowledge -education +choline
- (Neonate OR newborn) AND nutrition AND (gap OR deficiency) -rat -murine -mice -mouse -parenteral -enteral + choline
- (Infant OR baby) AND nutrition AND (gap OR deficiency) -parenteral -enteral +choline
- Toddler AND nutrition AND (gap OR deficiency) -parenteral -enteral choline
- Child AND nutrition AND (gap OR deficiency) -parenteral -enteral -knowledge -education +choline
- “First 1000 Days” AND (gap OR deficiency) + choline
- “First one thousand days” AND (gap OR deficiency) + choline
- (Pregnancy OR pregnant) AND (supplement OR supplementation) -knowledge -education +choline
- (Postnatal OR postpartum) AND (supplement OR supplementation) -knowledge -education -cows -cattle -heifer -dairy -mice -murine -rats -litter -weaning +choline
- (breast OR breastfed OR breastfeed OR breastfeeding) AND (supplement OR supplementation) -knowledge -education -cows -cattle -heifer -dairy -mice -murine -rats -litter -weaning +choline (Neonate OR newborn) AND (supplement OR supplementation) -knowledge -education -cows -cattle -heifer -dairy -mice -murine -rats -litter -weaning -parenteral -enteral +choline
- (Infant OR baby) AND (supplement OR supplementation) -parenteral -enteral +choline

- Toddler AND (supplement OR supplementation) -parenteral -enteral +choline
- Child AND (supplement OR supplementation) -parenteral -enteral -knowledge -education +choline
- First 1000 Days" AND (supplement OR supplementation) +choline
- Physiological Changes AND Pregnancy + Choline
- Nutrition AND 1st Trimester AND (2nd trimester or 3rd trimester) + Choline
- Nutrition AND Breastfeeding AND (human milk or formula) + Choline
- Nutrition AND Optimal Growth AND Development AND (newborn or infant or toddler) + Choline
- Healthy Mother AND Optimal Nutrition AND (Pregnancy OR Gestation OR Post-partum) + Choline
- Pregnancy AND (Gestation OR Post-partum) AND Nutrition AND (Gaps or Shortfall OR Deficiency) + Choline
- Healthy Child AND (Infant OR Toddler) AND Supplements AND (Prenatal OR Postnatal) + Choline
- Healthy Mother AND (Mom OR Parent) AND Supplements AND (Prenatal OR Postnatal) + Choline
- Microbiome AND (Microbiota OR Gut Bacteria) AND Pregnancy AND (Postpartum) + Choline
- Supplements AND (tablets OR dietary OR nutrition) AND Safety AND Pregnancy AND Postpartum + Choline
